# A Broad‐Spectrum ROS‐Eliminating Material for Prevention of Inflammation and Drug‐Induced Organ Toxicity

**DOI:** 10.1002/advs.201800781

**Published:** 2018-08-16

**Authors:** Lanlan Li, Jiawei Guo, Yuquan Wang, Xiaoxing Xiong, Hui Tao, Jin Li, Yi Jia, Houyuan Hu, Jianxiang Zhang

**Affiliations:** ^1^ Department of Pharmaceutics College of Pharmacy Third Military Medical University Chongqing 400038 China; ^2^ Department of Cardiology Southwest Hospital Third Military Medical University Chongqing 400038 China; ^3^ Department of Cardiology Affiliated Hospital of North Sichuan Medical College Nanchong 637000 Sichuan Province China; ^4^ Department of Neurosurgery Renmin Hospital of Wuhan University Wuhan 430060 China

**Keywords:** antioxidants, inflammation, nanoparticles, reactive oxygen species, targeted therapy

## Abstract

Despite the great potential of numerous antioxidants for pharmacotherapy of diseases associated with inflammation and oxidative stress, many challenges remain for their clinical translation. Herein, a superoxidase dismutase/catalase‐mimetic material based on Tempol and phenylboronic acid pinacol ester simultaneously conjugated β‐cyclodextrin (abbreviated as TPCD), which is capable of eliminating a broad spectrum of reactive oxygen species (ROS), is reported. TPCD can be easily synthesized by sequentially conjugating two functional moieties onto a β‐cyclodextrin scaffold. The thus developed pharmacologically active material may be easily produced into antioxidant and anti‐inflammatory nanoparticles, with tunable size. TPCD nanoparticles (TPCD NP) effectively protect macrophages from oxidative stress‐induced apoptosis in vitro. Consistently, TPCD NP shows superior efficacies in three murine models of inflammatory diseases, with respect to attenuating inflammatory responses and mitigating oxidative stress. TPCD NP can also protect mice from drug‐induced organ toxicity. Besides the passive targeting effect, the broad spectrum ROS‐scavenging capability contributes to the therapeutic benefits of TPCD NP. Importantly, in vitro and in vivo preliminary experiments demonstrate the good safety profile of TPCD NP. Consequently, TPCD in its native and nanoparticle forms can be further developed as efficacious and safe therapies for treatment of inflammation and oxidative stress‐associated diseases.

## 1 Introduction

Inflammation is a natural and protective response to different inciting stimuli such as injury, infection, trauma, toxins, and other imbalances.^1^ Generally, local and systemic inflammatory responses can resolve the infection, promote tissue repair and wound healing, and regain tissue homeostasis.^1^, ^2^, ^3^ However, uncontrolled, excessive acute, and chronic unresolved inflammation positively contributes to a large number of diseases, such as rheumatoid arthritis, chronic obstructive pulmonary disease, hepatitis, and diabetes as well as cardiovascular and neurodegenerative diseases.^4^, ^5^, ^6^ Consequently, anti‐inflammatory therapy has been considered to be a promising strategy for these diverse diseases.^4^, ^7^, ^8^ Historically, corticosteroids and nonsteroidal anti‐inflammatory drugs are widely used for the treatment of inflammatory diseases, but they may cause numerous side effects such as increased cardiovascular risk, osteoporosis, gastrointestinal bleeding, and chronic kidney disease.^9^, ^10^, ^11^ To overcome the drawbacks of these traditional small‐molecule drugs, biological therapeutics have been developed and clinically used, which can target specific inflammatory molecules, their receptors, or signaling pathways.^4^, ^12^ The representative anticytokine biologics include Infliximab (a chimeric anti‐TNF‐α monoclonal antibody), Etanercept (an anti‐TNF agent derived from TNF receptor‐p75 Fc fusion protein), and Anakinra (an interleukin‐1 receptor antagonist).^13^, ^14^ Despite their success, different limitations have been found for these anticytokine biologics. In addition to loss of response or developed intolerance in some patients,^15^ safety concerns such as serious infections and malignancies were observed in other cases.^16^, ^17^ The high cost and autoimmunity to antibodies also limit the clinical applications of these new biological therapies.

It has been well‐recognized that inflammation is intimately linked to oxidative stress.^18^ During an acute inflammatory response, the activated phagocytic cells such as granulocytes and macrophages produce high levels of reactive oxygen species (ROS) to kill the pathogens or digest foreign particles.^19^, ^20^ On the other hand, overproduction of ROS or continued oxidative stress can induce localized tissue injury or lead to chronic inflammation.^18^, ^21^, ^22^ Consequently, scavenging of ROS has been used as an antioxidative stress strategy for the treatment of numerous diseases associated with inflammatory disorders, in which a broad spectrum of antioxidants and radical scavengers are utilized. For example, Tempol (Tpl), a radical scavenger, and other small‐molecule antioxidants have been examined in a myriad of different animal models of diseases associated with local or systemic inflammation, such as paw edema,^23^ pancreatitis,^24^ colitis,^25^ atherosclerosis,^26^ and neurodegenerative diseases.^27^ Unfortunately, disappointing results have been reported by recent clinical trials on most antioxidants in treatment of chronic inflammatory diseases.^28^, ^29^ To a certain degree, this can be attributed to nonspecific distribution, rapid elimination via renal excretion, the poor permeability in specific tissues (such as the blood brain barrier), and the low retention at diseased sites (like atherosclerotic plaques).^30^, ^31^

To circumvent the abovementioned limitations of small‐molecule anti‐inflammatory and ROS‐scavenging agents, nanoparticles (NPs) derived from anti‐inflammatory materials have been investigated as new therapies for inflammation‐associated diseases.^30^, ^32^, ^33^, ^34^, ^35^ As a representative example, nanotherapies self‐assembled by Tempol‐containing amphiphilic copolymers were intensively examined for treatment of drug‐induced intestinal inflammation,^36^ nonalcoholic steatohepatitis,^37^ ischemia‐reperfusion injury,^38^ and radiation‐induced organ dysfunctions,^39^ affording remarkable success in animal studies. In addition, an azabisphosphonate‐capped dendrimer could serve as a nanotherapeutic to inhibit the development of inflammatory arthritis,^40^ while a dendrimer‐N‐acetyl‐l‐cysteine conjugate significantly suppressed neuroinflammation in newborn rabbits with cerebral palsy.^41^ Also, synthetic dendritic polyglycerol sulfates were able to dampen leukocyte extravasation in a dermatitis mouse model.^42^ Nanoparticles assembled by sugar‐based amphiphilic polymers were effective to counteract atherosclerosis, a chronic inflammatory disease.^43^ These studies have unambiguously demonstrated the effectiveness of anti‐inflammatory material‐derived nanotherapies. However, there are considerable barriers for their clinical translation. First, construction of these nanotherapies frequently requires polymers with relatively complicated chemical structures, but their structural tailoring, quality control, reproducible manufacturing, and/or validated characterization are still challenging for large‐scale production. Second, in vivo absorption, metabolism, and excretion profiles as well as the safety profile of these therapeutic nanoparticles remain to be fully addressed. The relatively high cost is an additional limiting factor. Thus far there is still unmet demand for the development of novel pharmacologically active materials for engineering of translational anti‐inflammatory nanotherapies.

Our previous studies demonstrated that a cyclic polysaccharide β‐cyclodextrin conjugated with oxidation‐labile units of phenylboronic acid pinacol esters can function as ROS‐responsive carrier materials for site‐specific release of diverse therapeutics.^44^, ^45^, ^46^, ^47^ Furthermore, these materials were able to eliminate hydrogen peroxide, thereby displaying antioxidative stress activity.^48^ Based on this promising finding and in view of the fact that the limited ROS‐eliminating capability by one single antioxidant may contribute to undesirable outcomes of most existing agents clinically studied,^28^ we hypothesize that functional materials with broad‐spectrum ROS‐scavenging capability are effective antioxidant and anti‐inflammatory agents for the management of diseases associated with the inflammatory response and oxidative stress injury. As a proof of concept, herein we report facile development of a β‐cyclodextrin material simultaneously linked with Tempol and phenylboronic acid pinacol ester. This material, in its native and nanoparticle forms, can efficiently eliminate multiple species of reactive oxygen, including superoxide anion, hydrogen peroxide, radical, and hypochlorite. In addition to in vitro experiments, we used four murine models to demonstrate in vivo efficacies of a nanotherapy based on the newly developed material, with respect to alleviating inflammation as well as drug‐induced organ toxicity. The in vivo safety profile of the used nanotherapy was also preliminarily substantiated after either intravenous or intraperitoneal (i.p.) administration at a high dose.

## 2 Results and Discussion

### 2.1 Design, Synthesis, and Characterization of a ROS‐Scavenging Material TPCD

**Figure 1:**
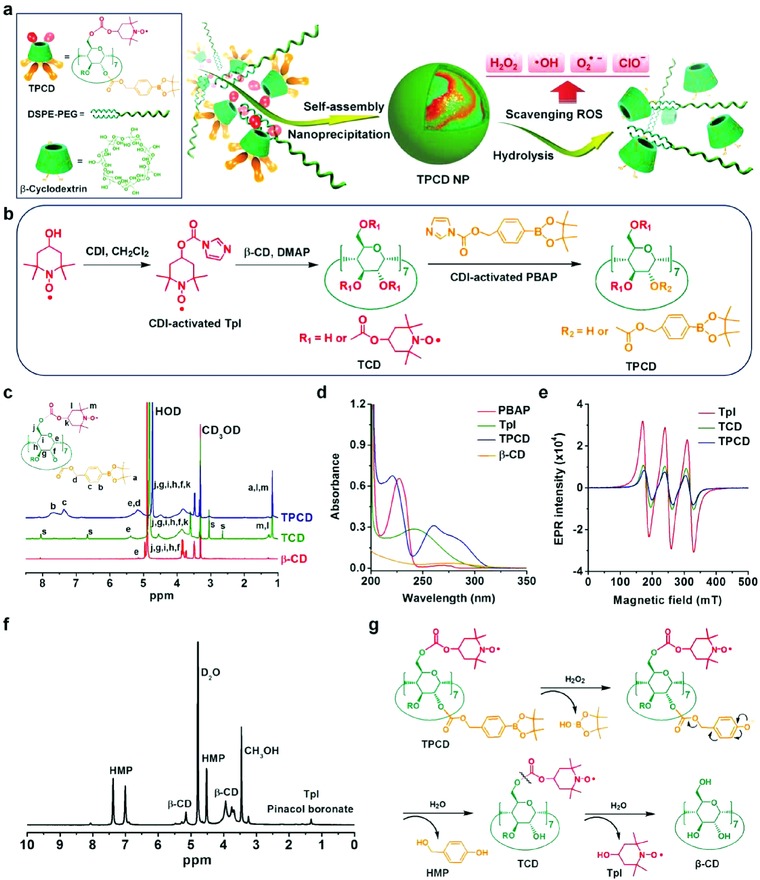
Design, preparation, and characterization of a ROS‐scavenging material. a) Schematic illustration of engineering of a broad spectrum ROS‐scavenging material and nanoparticle based on functionalized β‐cyclodextrin (β‐CD). b) The synthetic route of β‐CD conjugated with Tempol (Tpl) and PBAP units (TPCD). CDI, 1,1‐carbonyldiimidazole; DMAP, 4‐dimethylaminopyridine; TCD, Tpl‐conjugated β‐CD; PBAP, 4‐(hydroxymethyl) phenylboronic acid pinacol ester. c) ^1^H NMR spectra of different materials including β‐CD, TCD, and TPCD. The character “s” denotes the presence of trace amount of solvent impurities, such as DMSO and DMAP residuals from the precipitation process. d) UV–visible absorption spectra of different compounds. e) EPR spectra of free Tpl, TCD, and TPCD. f) A ^1^H NMR spectrum of the hydrolyzed TPCD sample in D_2_O. HMP, *p*‐(hydroxymethyl)phenol. g) The mechanism for the H_2_O_2_‐mediated hydrolysis of TPCD.

Tpl is a small‐molecule, superoxide dismutase (SOD)‐mimetic agent that can effectively scavenge superoxide anion and oxygen radicals.^49^ On the other hand, our previous study indicated that the phenylboronic acid pinacol ester (PBAP) group is able to stoichiometrically eliminate hydrogen peroxide (H_2_O_2_), thereby serving as a catalase‐mimetic moiety. According to these findings, we designed a pharmacologically functional material that can scavenge a broad spectrum of reactive species (**Figure** **1**a). This broad‐spectrum ROS scavenger, defined as TPCD, is derived from β‐cyclodextrin (β‐CD) simultaneously functionalized with Tpl and PBAP. We hypothesize that NPs based on TPCD are effective antioxidant and anti‐inflammatory nanotherapies for the treatment of diseases associated with oxidative stress and inflammation.

TPCD was synthesized by sequentially conjugating Tpl and PBAP onto β‐CD, which was achieved by activation of Tpl and PBAP with 1,1‐carbonyldiimidazole (CDI), followed by chemical conjugation of both moieties with hydroxyl groups of β‐CD (Figure 1b). According to our previous studies,^48^ PBAP‐conjugated β‐CD (PCD) materials can be totally hydrolyzed into water‐soluble products in the presence H_2_O_2_, regardless of the number of PBAP. In the case of Tpl‐conjugated β‐CD (TCD) materials, they were initially synthesized using excess amount of Tpl. Measurement by matrix‐assisted laser desorption/ionization time‐of‐flight (MALDI‐TOF) mass spectrometry revealed that TCDs are water‐soluble only when the conjugated Tpl unit is less than 4 (Figure S1, Supporting Information). In addition, TCDs cannot be effectively hydrolyzed in the presence of physiological levels of H_2_O_2_. Consequently, TCD with ≈2 Tpl units was used to synthesize TPCD that can be hydrolyzed into water‐soluble products in the presence of low levels of H_2_O_2_, which is beneficial for excretion from the body.

For the finally obtained TPCD, we found severely attenuated absorption at 3340 cm^−1^ due to the substitution of hydroxyl groups and appearance of evident signals from carbonyl (at 1735 cm^−1^), C—N stretch vibration (at 1430 cm^−1^), and phenyl groups (at 1680–1580 cm^−1^) in the Fourier transform infrared (FTIR) spectrum (Figure S2, Supporting Information). Consistently, ^1^H NMR spectroscopy confirmed the presence of Tpl and PBAP moieties in the resulting β‐CD material, in which proton signals from methyl of Tpl and phenyl groups of PBAP were clearly observed (Figure 1c). Also, the obtained product showed a UV‐visible spectrum with characteristic absorbance of both Tpl and PBAP (Figure 1d). The electron paramagnetic resonance (EPR) spectra of an intermediate compound TCD and the final material TPCD were similar to that of free Tpl (Figure 1e), which showed a sharp triplet signal attributable to the interaction between ^14^N and the unpaired electron of oxygen.^50^ Further characterization by MALDI‐TOF mass spectrometry affirmed the presence of covalently conjugated Tpl and PBAP units in TPCD (Figure S3a,b, Supporting Information). All these results demonstrated that TPCD was successfully synthesized. In addition, quantification of the hydrolyzed products by high‐performance liquid chromatography (HPLC) indicated that ≈2 Tpl and 5 PBAP units were covalently linked to each β‐CD molecule.

### 2.2 Hydrolysis Behaviors and ROS‐Scavenging Capability of TPCD

As demonstrated by previous studies, PBAP can be first oxidized to pinacol boronate and *p*‐quinone methide.^48^, ^51^, ^52^ Pinacol boronate ester is further hydrolyzed to pinacol and boric acid, while *p*‐quinone methide quickly turns into *p*‐(hydroxymethyl)phenol (HMP) in water. We analyzed hydrolysis products of TPCD by HPLC. At low concentrations of H_2_O_2_, hydrolysis of TPCD produced HMP after 24 h of incubation (Figure S4a, Supporting Information). In this case, only a small amount of Tpl was released (Figure S4b, Supporting Information). By contrast, exposure to high concentrations of H_2_O_2_ resulted in the generation of a considerable amount of both HMP and Tpl (Figure S4b,c, Supporting Information). This result indicated that hydrolysis of carbonate ester‐linked PBAP was more sensitive to H_2_O_2_ than that of Tpl. Measurements by ^1^H NMR spectroscopy and MALDI‐TOF mass spectrometry validated the production of β‐CD, Tpl, HMP, and pinacol boronate ester after complete hydrolysis of TPCD (Figure 1f and Figure S3c, Supporting Information). Consequently, under oxidative conditions, TPCD can be thoroughly hydrolyzed into water‐soluble products (Figure 1g), which can be easily eliminated from the body through the kidneys.

**Figure 2:**
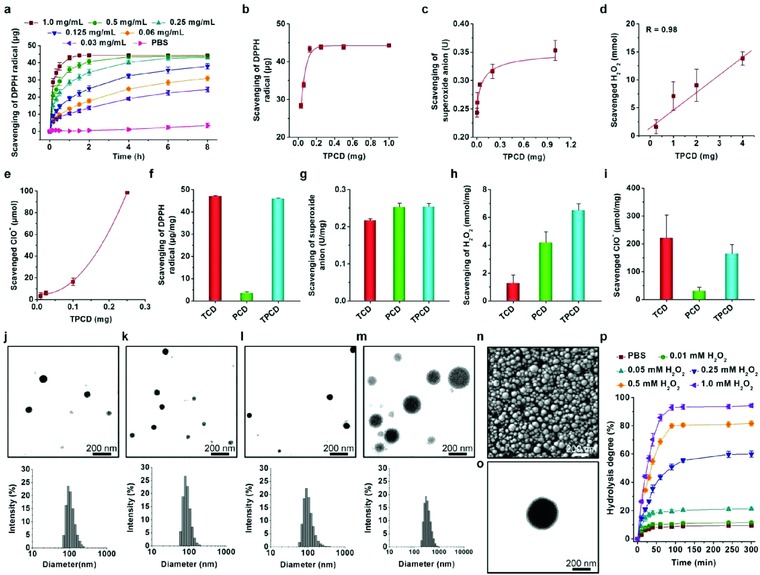
ROS‐scavenging capability of TPCD and characterization of TPCD nanoparticles. a) Time‐dependent scavenging of the DPPH radical by different doses of TPCD. b) DPPH radical‐scavenging efficiency of TPCD at varied doses after 20 h of incubation. c–e) Elimination of superoxide anion, H_2_O_2_, and hypochlorite by TPCD after incubation for 40 min, 24 h, and 15 min, respectively. f–i) Comparison of scavenging capabilities of different materials for radical, superoxide anion, H_2_O_2_, and hypochlorite. j–m) TEM images (the upper panel) and size distribution profiles (the lower panel) of TPCD NPs fabricated by a self‐assembly/nanoprecipitation method using methanol, methanol/acetonitrile, methanol/dimethylformamide, and methanol/tetrahydrofuran as the solvent for TPCD, respectively. For all solvent mixtures, the volume ratio of methanol to another solvent was kept at 1:2. n) A typical SEM image of TPCD NP prepared using methanol as the solvent. o) TEM image of phosphotungstic acid‐stained TPCD NP. p) Hydrolysis curves of TPCD NP (prepared using methanol as a solvent) in PBS (0.01 m, pH 7.4) containing various concentrations of H_2_O_2_. Data in (a–i, p) are mean ± SE (*n* = 3).

The ROS‐scavenging capacity of TPCD was then examined in vitro. First, we evaluated the free radical eliminating capability of TPCD by 2,2‐diphenyl‐1‐picrylhydrazyl (DPPH) assay.^39^ In the absence of TPCD, the DPPH radical was stable during the examined time period of 8 h (**Figure** **2**a). Upon incubation with TPCD, a dose and time‐dependent elimination profile was observed. At 1.0 mg mL^−1^ of TPCD, 2 h of incubation led to nearly 90% scavenging of the DPPH radical. After 30 min of incubation, the DPPH median elimination concentration (EC_50_) was 0.85 mg mL^−1^. It was also found that the radical eliminating capacity was proportional to the dose of TPCD (Figure 2b). Using commercially available kits, we determined the elimination of superoxide anion (O_2_·^−^) and H_2_O_2_ by TPCD. As expected, TPCD was able to effectively scavenge superoxide anion and H_2_O_2_, in a dose‐response pattern after incubation for 40 min and 24 h, respectively (Figure 2c,d). To quantify the hypochlorite (ClO^−^) elimination capacity of TPCD, we used a luminescent nanoprobe developed in our previous study (Figure S5, Supporting Information).^53^ The result indicated that hypochlorite can be efficiently consumed by TPCD, displaying a dose‐dependent profile after 15 min of incubation (Figure 2e). Compared with TCD or PCD alone, only TPCD can efficiently scavenge all the examined reactive species (Figure 2f–i). Collectively, these results demonstrated that TPCD was capable of eliminating a broad spectrum of reactive species.

### 2.3 Preparation and Characterization of TPCD Nanoparticles

TPCD NPs were produced by a modified nanoprecipitation/self‐assembly method in the existence of a small amount of lecithin and DSPE‐PEG.^44^ Since methanol is a good solvent for the synthesized TPCD examined in this study, it was used during the preparation of NPs based on TPCD. When methanol was used to dissolve TPCD, spherical NPs with a relative narrow size distribution profile were obtained, as illustrated by transmission electron microscopy (TEM) images (Figure 2j). Measurement by dynamic light scattering (DLS) revealed a mean diameter of 109 ± 2 nm. The zeta potential was −16 ± 0.1 mV. Notably, the size of TPCD NPs could be regulated by using different solvent combinations during preparation. For example, negatively charged TPCD NPs with diameters of 78 ± 5, 108 ± 11, and 372 ± 10 nm were successfully fabricated when solvent mixtures of methanol/acetonitrile, methanol/dimethylformamide, and methanol/tetrahydrofuran were used, respectively (Figure 2k–m and Figure S6, Supporting Information). Characterization by scanning electron microscopy (SEM) confirmed the spherical shape of TPCD NP thus prepared (Figure 2n). In addition, TEM observation of phosphotungstic acid‐stained samples revealed a core–shell structure for thus obtained TPCD NP (Figure 2o). In the following experiments, TPCD NP prepared using methanol as a solvent was used.

Subsequently, in vitro hydrolysis tests were performed to examine ROS‐sensitivity of TPCD NP. It was found that the hydrolysis rate and degree of TPCD NP were positively correlated to the H_2_O_2_ concentration (Figure 2p). At low concentrations of H_2_O_2_, slow hydrolysis was observed after 5 h of incubation. By contrast, both hydrolysis rate and hydrolysis degree were considerably increased when TPCD NP was separately incubated with H_2_O_2_ at 0.25, 0.5, and 1.0 × 10^−3^ m. After 90 min of incubation, a hydrolysis percentage of 93% was found at 1.0 × 10^−3^ m H_2_O_2_. This hydrolysis profile of TPCD NP is consistent with the ROS‐responsive nature of its native material TPCD, demonstrating that the oxidation‐sensitivity was well‐maintained after processing into TPCD NP. This also demonstrated our design principle that TPCD can be hydrolyzed into water‐soluble products when less than 3 Tpl units are conjugated. Notably, TPCD NP displayed negligible hydrolysis in PBS without H_2_O_2_. In this case, less than 8% TPCD NP was hydrolyzed. In separate experiments, the H_2_O_2_ concentration‐dependent hydrolysis of TPCD NP was examined by DLS. After 3 h of incubation in PBS with different concentrations of H_2_O_2_, a significant decrease in particle size was found (Figure S7a,b, Supporting Information), with the decrease degree positively correlated to the H_2_O_2_ concentration. Concomitantly, the zeta‐potential value was evidently reduced after hydrolysis (Figure S7c, Supporting Information). Further observation by TEM also confirmed the H_2_O_2_‐dependent hydrolysis profile of TPCD NP (Figure S7d, Supporting Information). The smallest particle size was observed for TPCD NP incubated at 1.0 × 10^−3^ m H_2_O_2_, suggesting dramatic hydrolysis in this case, which well agrees with the DLS result. Since the maximal extracellular H_2_O_2_ level in normal mammalian tissues is within the range of 2–4 × 10^−6^ m,^54^ a minimal amount of TPCD NP would be hydrolyzed in healthy tissues, thereby diminishing possible side effects.

### 2.4 Cellular Uptake and In Vitro Antioxidative Stress Activity of TPCD NP in Macrophages

The generation of intracellular ROS is one of the key features of inflammatory cells.^18^ The regulation of intracellular redox homeostasis is an important strategy to attenuate oxidative stress‐induced cell death and tissue injury.^18^, ^55^ Accordingly, we examined cellular internalization of TPCD NP in macrophages because of their critical role in the pathogenesis of numerous inflammatory diseases. After Cy5‐labeled TPCD NP was incubated with RAW264.7 mouse macrophage cells, cellular uptake was evaluated by fluorescence microscopy in combination with flow cytometry.

**Figure 3:**
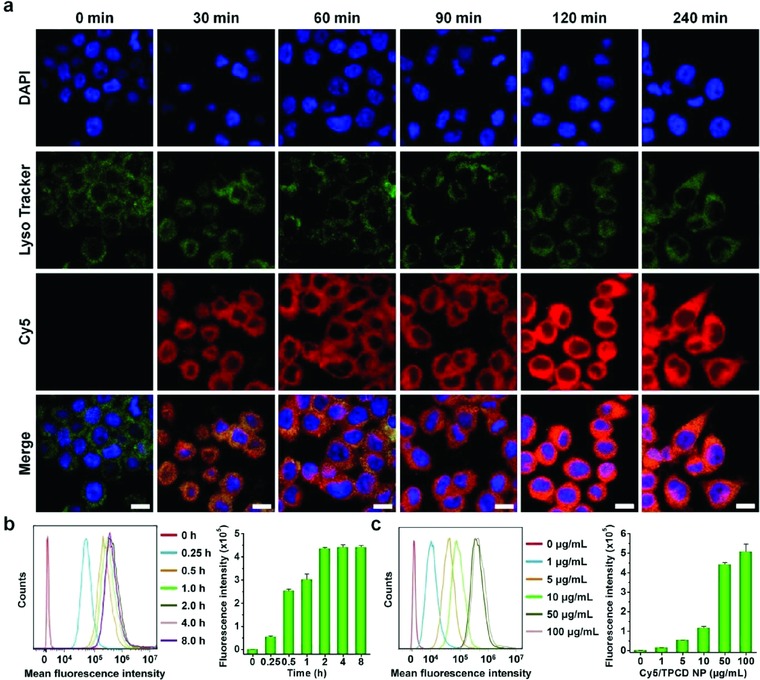
Cellular uptake of Cy5‐labeled TPCD NP in RAW264.7 macrophages. a) Fluorescence images showing time‐dependent internalization of Cy5/TPCD NP at 2 µg mL^−1^ of Cy5 in RAW264.7 cells. For observation by confocal microscopy, nuclei were stained with DAPI (blue), while late endosomes and lysosomes were stained with LysoTracker (green). Scale bars, 10 µm. b) Typical flow cytometric curves (left) and quantitative analysis (right) of time‐dependent cellular uptake of Cy5/TPCD NP at 2 µg mL^−1^ of Cy5 in RAW264.7 cells. c) Flow cytometric profiles (left) and quantification results (right) indicating cellular uptake of Cy5/TPCD NP at various doses after 2 h of incubation in RAW264.7 cells. Data are mean ± SE (*n* = 3).

Observation by confocal microscopy showed time‐dependent internalization of Cy5/TPCD NP in RAW264.7 cells (**Figure** **3**a). Even after 30 min of incubation, significant red fluorescence was found in cells. The fluorescent signals of Cy5/TPCD NP were significantly increased with prolonged incubation. Moreover, colocalization of Cy5 fluorescence with green fluorescence due to LysoTracker was clearly observed. Since the fluorescence of acidotropic dye LysoTracker was mainly derived from late endosomes and lysosomes, this result indicated that internalized Cy5/TPCD NP was transported via the endolysosomal pathway in RAW264.7 cells. Consistent with the finding based on fluorescent observation, quantification by flow cytometry showed gradually intensified fluorescent signals in RAW264.7 cells when the incubation time was increased (Figure 3b). Also, flow cytometric analysis revealed a dose‐dependent endocytosis of Cy5/TPCD NP (Figure 3c). Together, these results demonstrated that TPCD NP can be rapidly and efficiently internalized by macrophages.

**Figure 4:**
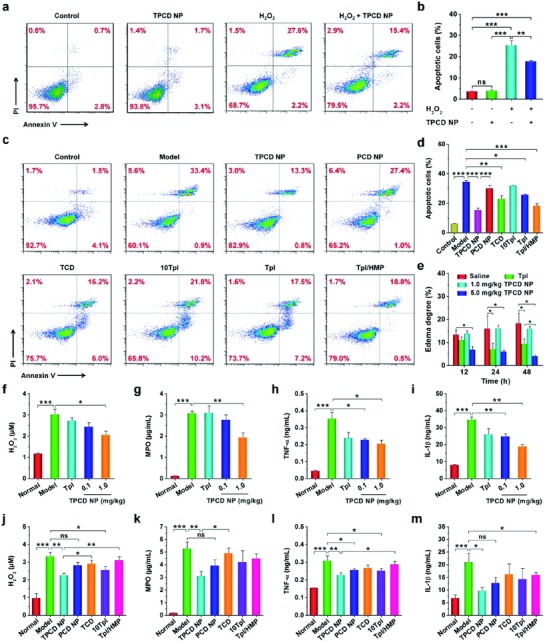
In vitro antioxidative stress and in vivo anti‐inflammatory activities in murine models of acute inflammation. a,b) Flow cytometric profiles and quantitative data of apoptotic RAW264.7 cells after different treatments. The control, TPCD NP, and H_2_O_2_ groups were treated with fresh medium, 100 µg mL^−1^ of TPCD NP, and 200 × 10^−6^ m H_2_O_2_, respectively. The H_2_O_2_ + TPCD NP group was first incubated with 100 µg mL^−1^ of TPCD NP for 2 h, followed by culture with 200 × 10^−6^ m H_2_O_2_ for 24 h. c,d) Flow cytometric profiles and quantitative analysis of apoptotic macrophages treated with different formulations. The groups of TPCD NP, PCD NP, TCD, 10Tpl, Tpl, and Tpl/HMP were preincubated with the corresponding formulations for 2 h, and then stimulated with 200 × 10^−6^ m H_2_O_2_ for 24 h. The 10Tpl group was treated with Tpl at the tenfold dose of that contained in TPCD NP. e) In vivo efficacy of TPCD NP in rats with carrageen‐induced edema. At 0.5 h after challenge by i.d. injection of carrageen, a single dose of different formulations was locally administered. The dose of free Tpl was the same as that of TPCD NP at 5.0 mg kg^−1^. f–i) The levels of H_2_O_2_, MPO, TNF‐α, and IL‐1β in cell‐free peritoneal exudates collected from mice with zymosan‐induced peritonitis. At 1 h after zymosan induction by i.p. injection, different treatments were performed. TPCD NP at 1.0 mg kg^−1^ contained the same dose of Tpl as the free Tpl group. j–m) The expression of H_2_O_2_, MPO, TNF‐α, and IL‐1β in peritoneal exudates from peritonitis mice after treatment with different controls. Data are mean ± SE (b, d, *n* = 3; e, *n* = 5; f–m, *n* = 6). Statistical significance was assessed by one‐way ANOVA with post hoc LSD tests for data in (b, e–m). *P < 0.05, **P < 0.01, ***P < 0.001; ns, no significance. Due to heterogeneity of variance, the Kruskal–Wallis test was used for statistical analysis of data in (d). *P < 0.001, **P < 0.0001, ***P < 0.00001.

We then investigated whether TPCD NP is capable of protecting macrophages from ROS‐induced apoptosis. Exposure of RAW264.7 cells to 200 × 10^−6^ m H_2_O_2_ resulted in significant apoptosis when compared with the control cells treated with fresh medium, as illustrated by flow cytometric analysis (**Figure** **4**a,b). By contrast, treatment with 100 µg mL^−1^ TPCD NP significantly attenuated H_2_O_2_‐mediated cell apoptosis. Of note, incubation of unstimulated RAW264.7 cells with TPCD NP alone did not cause significant apoptosis, which was comparable to those treated with fresh medium. In a separate study, PCD NP and TCD were used as β‐CD derived controls with relatively narrow‐spectrum ROS‐eliminating capacity. In addition, a high dose of Tpl and combined use of Tpl/HMP served as small‐molecule controls. HMP was used since previous studies have demonstrated that HMP itself displays anti‐inflammatory and antioxidative stress activities in animal models,^33^, ^56^ while hydrolysis of TPCD can generate HMP. For the 10Tpl group, the Tpl dose was tenfold of that contained in TPCD NP. In the Tpl/HMP group, the Tpl dose was the same as that in TPCD NP, while the dose of HMP equaled to that produced after complete hydrolysis of TPCD NP. In this case, the lowest apoptosis was found for macrophages treated with TPCD NP (Figure 4c,d), although varied degrees of decrease in apoptosis were also observed in other groups. Of note, high‐dose Tpl even showed no significant inhibitory activity as compared to low‐dose Tpl. Consequently, TPCD NP can significantly inhibit oxidative stress‐mediated cell apoptosis by effectively scavenging ROS, which displayed more desirable effects than therapies with narrow‐spectrum ROS‐eliminating capacity.

### 2.5 Therapeutic Effects of TPCD NP in Animal Models of Inflammatory Diseases

Based on the above promising results, in vivo studies were performed to examine therapeutic benefits of the newly engineered ROS‐scavenging nanotherapy in different animal models of inflammatory diseases, in view of the fact that ROS‐mediated oxidative stress is intimately associated with both acute and chronic inflammation.^18^, ^57^ All the animal care and experimental protocols were performed in compliance with the Animal Management Rules of the Ministry of Health of the People's Republic of China (No. 55, 2001) and the guidelines for the Care and Use of Laboratory Animals of Army Medical University (Chongqing, China). First, we employed a rat model of paw edema induced by intradermal (i.d.) injection of carrageen, which has been widely used for screening of anti‐inflammatory drugs.^58^ Different treatments were also performed by i.d. administration. Compared with the diseased rats treated with saline, the degrees of paw edema were significantly inhibited by treatment with 5.0 mg kg^−1^ of TPCD NP, as examined at 12, 24, and 48 h after stimulation with carrageen (Figure 4e). Nevertheless, we did not find beneficial effects when a relatively low dose of 1.0 mg kg^−1^ was administered. Of note, therapy with 5.0 mg kg^−1^ TPCD NP was more effective than the same dose of free Tpl. This result implied that TPCD NP displayed anti‐inflammatory activity, in a dose–response pattern.

Subsequently, another well‐recognized acute inflammation model of peritonitis was used to evaluate in vivo anti‐inflammatory efficacy of TPCD NP. Peritonitis in mice was induced by i.p. injection of zymosan.^53^ At 6 h after initiation of inflammation, the levels of both oxidative stress‐associated molecular mediators (H_2_O_2_ and myeloperoxidase, i.e., MPO) and typical pro‐inflammatory cytokines (TNF‐α and IL‐1β) in cell free peritoneal lavage fluid were significantly increased in the model group treated with the saline (Figure 4f–i). By contrast, these pro‐inflammatory mediators, particularly TNF‐α and IL‐1β were effectively decreased by local treatment with TPCD NP at 0.1 mg kg^−1^. Furthermore, the antioxidant and anti‐inflammatory activities of TPCD NP were notably increased when its dose was enhanced to 1.0 mg kg^−1^, as demonstrated by the significantly reduced levels of MPO, H_2_O_2_, TNF‐α, and IL‐1β. Notably, treatment with free Tpl at the same dose of 1.0 mg kg^−1^ TPCD NP showed efficacy comparable to that of 0.1 mg kg^−1^ TPCD NP.

Also, we compared in vivo efficacies using different nanotherapy and small‐molecule controls as aforementioned in apoptosis studies. Quantification of typical mediators related to oxidative stress and inflammation (including H_2_O_2_, MPO, TNF‐α, and IL‐1β) in peritoneal lavage fluid revealed varied degrees of efficacies after peritonitis‐bearing mice were treated with PCD NP, TCD, tenfold Tpl, and Tpl/HMP (Figure 4j–m). For all the examined mediators, the TPCD NP group showed significant difference as compared to the model group. By contrast, only some of the detected mediators exhibited statistical significance after therapy with other formulations. Overall, more desirable effects were achieved after treatment with TPCD NP, consistent with the finding of in vitro cell culture experiments. Taken together, these results demonstrated that TPCD NP displayed potent anti‐inflammatory activity by effectively attenuating oxidative stress at the disease sites of acute inflammation.

### 2.6 Therapy of Acute Lung Injury in Mice with TPCD NP

**Figure 5:**
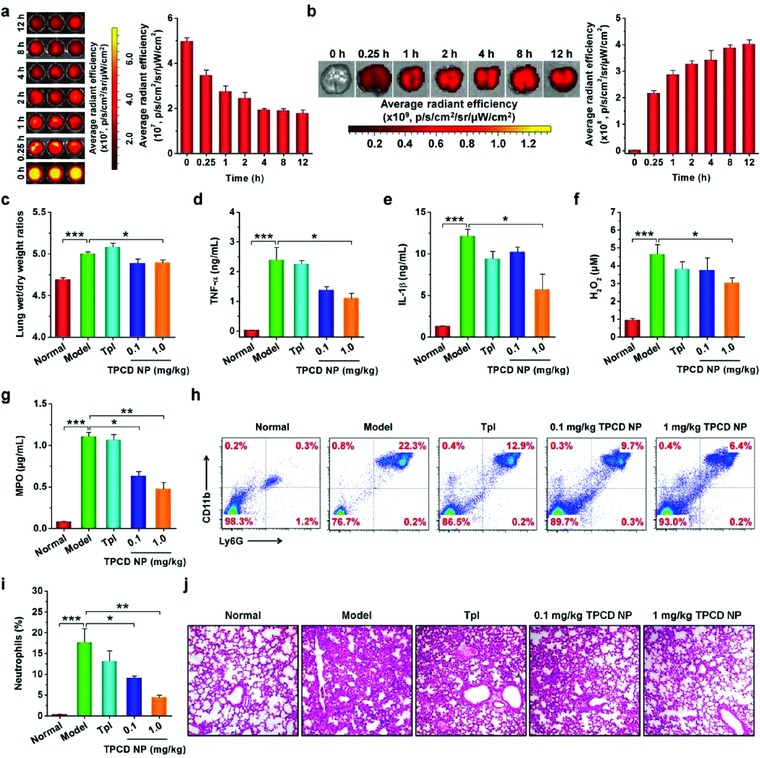
Targeted therapy of acute lung injury with TPCD NP in mice. a) Fluorescence images (left) and quantitative analysis (right) of the blood samples collected from mice with acute lung injury (ALI) at various time points after i.v. injection of Cy7.5/TPCD NP. b) Ex vivo images (left) and quantitative analysis (right) illustrating the distribution of Cy7.5/TPCD NP in the lung of ALI mice after i.v. administration for different periods of time. c) The lung wet‐to‐dry weight ratios after different treatments. d–g) The expression levels of TNF‐α, IL‐1β, H_2_O_2_, and MPO in bronchoalveolar lavage fluid from mice with LPS‐induced ALI and subjected to different treatments. h,i) Representative flow cytometric profiles and quantitative analysis of neutrophil counts in pulmonary tissues of ALI mice. j) H&E‐stained pathological sections of lung tissues. ALI in mice was induced by intratracheal (i.t.) administration of LPS. 1 h after challenge, mice were i.v. administered with different formulations. The normal group was treated with saline. The TPCD NP groups were administered with either lower (0.1 mg kg^−1^) or higher (1.0 mg kg^−1^) dose of TPCD NP, and 1.0 mg kg^−1^ TPCD contained the same dose of the Tpl unit as the free Tpl group. At 12 h after various treatments, bronchoalveolar lavage fluid was collected for different quantitative analyses. In separate experiments, lung tissues were isolated for additional quantitative and histological analyses. Data are mean ± SE (a,b, *n* = 3; c–g, i, *n* = 6). Statistical significance was assessed by one‐way ANOVA with post hoc LSD tests. *P < 0.05, **P < 0.01, ***P < 0.001.

Then we evaluated therapeutic benefits of TPCD NP in mice with induced acute lung injury (ALI), an inflammatory pulmonary disease with the most severe manifestation of acute respiratory distress syndrome, which remains a significant source of morbidity and mortality in critically ill patients.^59^ ALI is closely related to both oxidative stress and inflammatory responses.^59^, ^60^ According to a previously established method,^53^ ALI in mice was induced by intratracheal (i.t.) instillation of lipopolysaccharide (LPS). Before therapeutic assessments, we first interrogated whether TPCD NP can be accumulated at the injured lung tissue. After i.v. administration of Cy7.5‐labeled TPCD NP in mice with ALI, we observed a relatively rapid clearance profile from the bloodstream, with a half‐life of 2.3 h (**Figure** **5**a). By contrast, the fluorescent signals of Cy7.5 were rapidly increased in the lungs of ALI mice (Figure 5b). Even at 15 min after i.v. injection, significant Cy7.5 fluorescence was observed. This result demonstrated that TPCD NP can effectively target to injured lungs in mice, and the accumulated NPs were maintained at the injured tissues for a relatively long period of time. In addition to accumulation in the lung, distribution of Cy7.5/TPCD NP in other organs such as heart, liver, spleen, and kidney was observed (Figure S8a, Supporting Information). Quantitative analysis revealed dramatically increased fluorescent signals in heart even at 0.25 h after i.v. administration, while the fluorescent intensities were gradually enhanced in other organs (Figure S8b, Supporting Information). Nevertheless, the fluorescent signals in heart were significantly lower than those in other organs examined. Subsequently, we compared targeting efficiency of i.v. administered Cy7.5/TPCD NP in lungs of normal and diseased mice. Whereas accumulation in the normal lung was observed at 12 h after i.v. injection, significantly higher fluorescence was found in the injured lung (Figure S9, Supporting Information), which can be attributed to the enhanced permeability due to disruption of the lung endothelial and epithelial barriers.^59^ Nevertheless, independent of healthy or diseased mice, similar distribution profiles were found in other major organs such as heart, liver, spleen, and kidney (Figure S10, Supporting Information).

Subsequently, in vivo therapeutic effects of TPCD NP were examined in mice with ALI. In the model group treated with saline, the lung wet/dry weight ratio, an index of pulmonary edema due to acute inflammation,^61^ was significantly increased (Figure 5c). This parameter was notably decreased after treatment with TPCD NP at either 0.1 or 1.0 mg kg^−1^ by i.v. injection. By contrast, treatment with free Tpl at a dose equals to 1.0 mg kg^−1^ TPCD NP showed no significant effects. Also, the pro‐inflammatory cytokines TNF‐α and IL‐β in bronchoalveolar lavage fluid were remarkably reduced after treatment with TPCD NP, particularly at 1.0 mg kg^−1^ (Figure 5d,e). Moreover, the ROS level and MPO expression were significantly decreased by therapy with 1.0 mg kg^−1^ TPCD NP (Figure 5f,g). Since acute inflammation and oxidative stress‐induced injury are mechanistically associated with neutrophils,^62^ we detected their infiltration in lungs. The saline‐treated, diseased mice showed a considerably higher neutrophil count in bronchoalveolar lavage fluid as compared to that of the normal mice (Figure 5h,i). Treatment with TPCD NP at either 0.1 or 1.0 mg kg^−1^ significantly suppressed neutrophil infiltration. This is also in line with the decreased MPO level, because MPO is generally expressed by activated neutrophils. Further evaluation was performed by examination on hematoxylin and eosin (H&E)‐stained sections. The model group displayed significant infiltration of inflammatory cells and alveolar hemorrhage (Figure 5j), which are characteristic patterns for ALI. These histological abnormalities were significantly mitigated by treatment with the ROS‐scavenging nanotherapy TPCD NP. Therapy with Tpl, however, did not afford significant therapeutic outcome (Figure 5c–j). On the one hand, this is attributed to nonspecific distribution of Tpl after i.v. administration, causing a low localization of Tpl molecules at diseased sites. On the other hand, Tpl can only eliminate part of reactive species.

**Figure 6:**
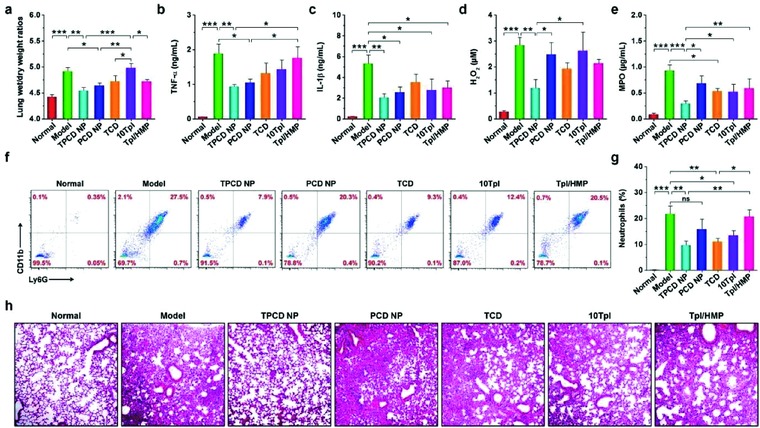
Comparison of in vivo efficacies of TPCD NP with different nanotherapy and small‐molecule controls in ALI mice. a) The lung wet‐to‐dry weight ratios after treatment with different formulations. b–e) The levels of TNF‐α, IL‐1β, H_2_O_2_, and MPO in bronchoalveolar lavage fluid from mice with LPS‐induced ALI and subjected to different treatments. f,g) Flow cytometric profiles and quantitative data of neutrophil counts in pulmonary tissues of ALI mice. h) H&E‐stained sections of lung tissues. ALI in mice was induced by intratracheal (i.t.) administration of LPS. After 1 h, mice were treated with different formulations via i.v. injection. The normal group was treated with saline. The PCD NP and TCD groups were administered at the same dose of the PBAP or Tpl unit as that of 1.0 mg kg^−1^ of TPCD NP, respectively. In the 10Tpl group, the Tpl dose was tenfold of that contained in TPCD NP. For the Tpl/HMP group, the Tpl dose was the same as that in TPCD NP, while the HMP dose equaled to that generated after complete hydrolysis of TPCD NP. At 12 h after different treatments, bronchoalveolar lavage fluid was collected for quantitative analyses. In a separate study, lung tissues were isolated for quantitative and histological analyses. Data are mean ± SE (a–e, g, *n* = 6). Statistical significance was assessed by one‐way ANOVA with post hoc LSD tests. *P < 0.05, **P < 0.01, ***P < 0.001; ns, no significance.

Moreover, therapeutic effects of TPCD NP were also compared with those of PCD NP, TCD, high‐dose Tpl, and Tpl/HMP. Compared with the model group, TPCD NP afforded significant differences, with respect to decreasing lung wet/dry weight ratios as well as reducing the expression levels of TNF‐α, IL‐1β, H_2_O_2_, and MPO in bronchoalveolar lavage fluid (*P* < 0.01 or *P* < 0.001, **Figure** **6**a–e). Whereas treatment with PCD NP also significantly reduced lung wet/dry weight ratios, TNF‐α, and IL‐1β (*P* < 0.05), no beneficial outcomes were found in inhibition of H_2_O_2_ and MPO (*P* > 0.05). Likewise, limited effects were obtained for TCD, 10‐fold Tpl, and Tpl/HMP. This was further affirmed by the significantly reduced neutrophil count and notably improved lung tissue structure as manifested by H&E sections (Figure 6f–h). Taken together, by targeting the injured lungs and effectively scavenging a broad spectrum of ROS, TPCD NP can function as an efficacious nanotherapy for the treatment of ALI.

### 2.7 Treatment of Drug‐Induced Liver and Kidney Toxicity with TPCD NP

**Figure 7:**
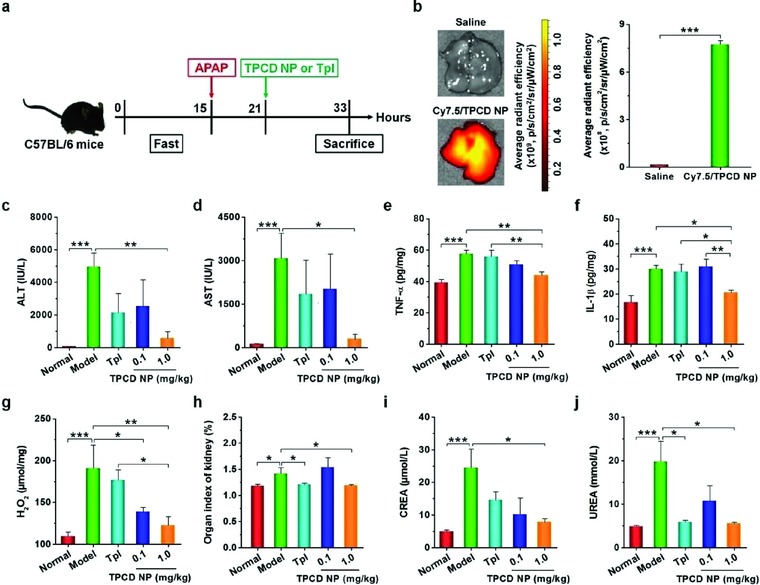
Detoxification of acetaminophen (APAP)‐induced hepatotoxicity and renal injury by TPCD NP. a) Schematic illustration of the establishment of APAP‐induced organ toxicity and treatment regimens. b) Representative ex vivo images and quantitative analysis of Cy7.5/TPCD NP accumulation in the liver of mice at 12 h after administration. c,d) The levels of ALT and AST in serum collected from mice with APAP‐induced toxicity. e–g) The expression levels of TNF‐α, IL‐1β, and H_2_O_2_ in the hepatic tissues. h) The organ index of kidney. i,j) The serum levels of CREA and UREA. For therapy studies, at 6 h after i.p. stimulation with APAP at 200 mg kg^−1^, mice were treated with different formulations. Mice in the normal group were not challenged with APAP. The model group was administered with saline. TPCD NP at 1.0 mg kg^−1^ contained the same dose of the Tpl unit as that of the free Tpl group. At 12 h after different treatments, animals were euthanized and serum was collected for quantification of biochemical markers. Additionally, the liver tissues were isolated for quantification of inflammatory mediators. Data are mean ± SE (b, *n* = 3; c–j, *n* = 6). Statistical significance was analyzed by one‐way ANOVA with post hoc LSD tests. *P < 0.05, **P < 0.01, ***P < 0.001.

We also examined therapeutic benefits of TPCD NP in acute liver and kidney injury induced by acetaminophen (APAP, an analgesic and antipyretic drug), which is intimately related to oxidative stress due to the overproduction of ROS.^63^ Mice were stimulated by i.p. injection of APAP (**Figure** **7**a). After i.v. administration, we observed efficient accumulation of Cy7.5/TPCD NP in the liver, as indicated by the strong fluorescent signal of Cy7.5 (Figure 7b). Of note, we found significantly higher accumulation of Cy7.5/TPCD NP in the injured liver than that of the healthy liver from normal mice (Figure S11, Supporting Information). Likewise, Cy7.5 fluorescent signals were detected in other organs (including heart, spleen, lung, and kidney), exhibiting similar distribution profiles in both healthy and injured mice (Figure S12, Supporting Information). This implied that i.v. delivered TPCD NP can passively target the liver, particularly the liver with induced injury. Our result is consistent with previous findings on animals with or without liver diseases.^64^, ^65^

Then we interrogated in vivo efficacy of TPCD NP after i.v. delivery at 6 h after APAP challenge in mice (Figure 7a). Significantly higher levels of serum alanine aminotransferase (ALT) and aspartate aminotransferase (AST) were detected for the model mice (Figure 7c,d), which are generally observed for APAP‐induced hepatotoxicity.^66^ Treatment with i.v. administered TPCD NP decreased both ALT and AST, in a dose‐dependent manner, with the statistically significant efficacy achieved at 1.0 mg kg^−1^. By contrast, free Tpl at a dose corresponding to 1.0 mg kg^−1^ TPCD NP did not significantly reduce ALT and AST. Consistently, therapy with 1.0 mg kg^−1^ of TPCD NP notably inhibited the abnormally increased expression of pro‐inflammatory cytokines TNF‐α and IL‐1β (Figure 7e,f). We also detected remarkably decreased ROS levels after TPCD NP treatment (Figure 7g). Treatment with free Tpl, however, showed no significant effects on the levels of TNF‐α, IL‐1β, and ROS. Similarly, therapy with TPCD NP effectively mitigated APAP‐induced nephrotoxicity, as manifested by notably alleviated hydronephrosis and significantly decreased serum levels of creatinine (CREA) and urea (UREA) (Figure 7h–j). As well documented, both CREA and UREA are markers of acute renal failure.^67^ In this case, treatment with the same dose of Tpl also resulted in a certain degree of efficacy, as indicated by the significantly reduced kidney index and UREA.

**Figure 8:**
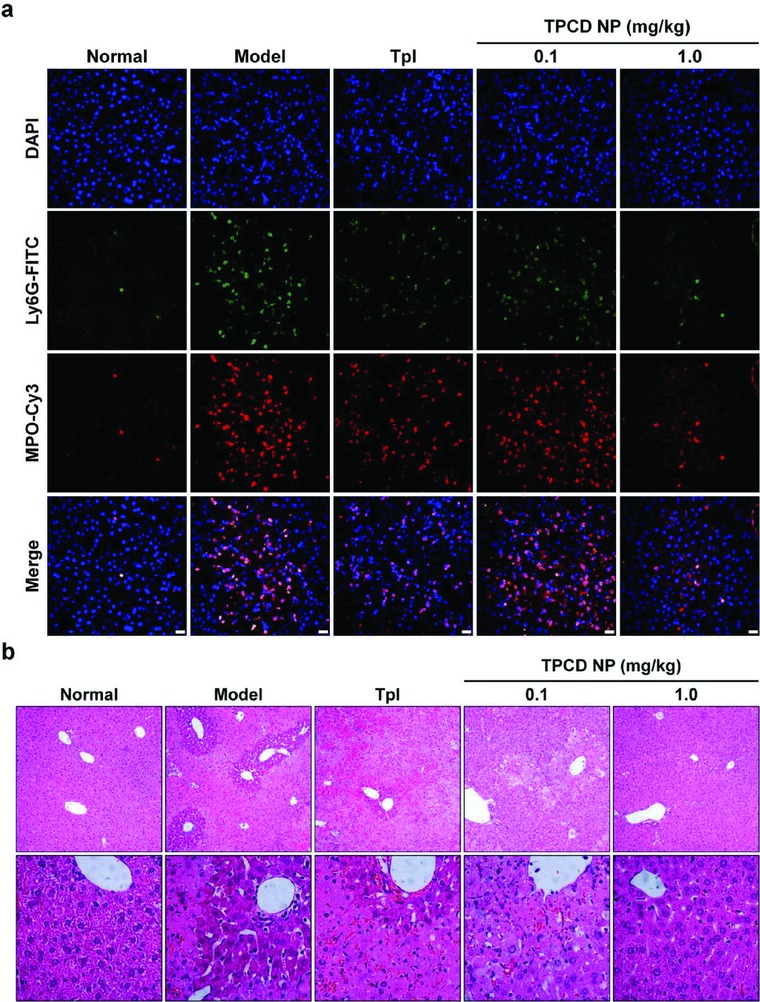
Evaluation of in vivo detoxification efficacy of TPCD NP in mice with APAP‐induced hepatotoxicity. a) Immunofluorescence images show the infiltration of neutrophils and expression of MPO in the liver. Scale bars, 20 µm. b) H&E‐stained histopathological sections of liver tissues resected from mice subjected to different treatments. The lower panel indicates high‐resolution images.

Further detections were performed to examine the therapeutic outcome in the case of liver injury. Immunofluorescence analysis indicated dramatically high infiltration of neutrophils in liver sections of the model group (**Figure** **8**a), consistent with the fact that neutrophils are frequently involved in the pathogenesis of acute liver toxicity.^68^ After treatment with different formulations, the neutrophil count was considerably decreased, especially for 1.0 mg kg^−1^ TPCD NP. In line with the inhibited neutrophil infiltration, the expression of MPO was also significantly inhibited by treatment with TPCD NP at 1.0 mg kg^−1^. Additionally, histological examination revealed considerable necrotic cell death of hepatocytes and cell vacuolization as well as significant injury/necrosis of sinusoidal endothelial cells and local hemorrhage in the model group (Figure 8b). Whereas these histological abnormalities were partly reversed by treatment with Tpl, a more efficacious effect was attained by the same dose of TPCD NP. The histological structures of sections from the mice treated with 1.0 mg kg^−1^ TPCD NP were even comparable to those of the normal mice treated with saline.

**Figure 9:**
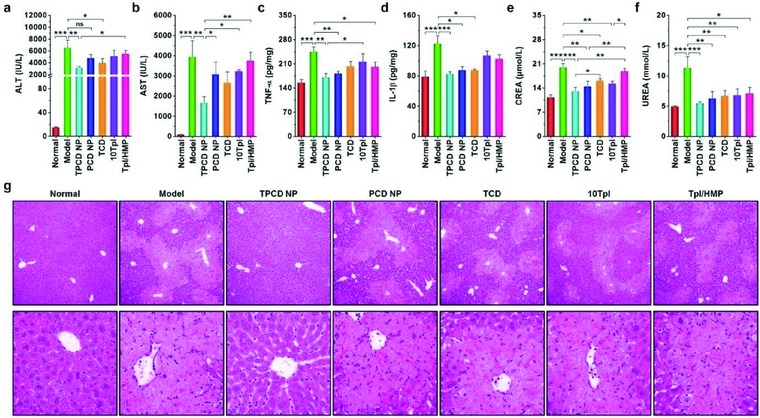
Comparison of in vivo detoxification efficacy of TPCD NP with different nanotherapy and small‐molecule controls in mice with APAP‐induced hepatotoxicity and renal injury. a,b) The levels of ALT and AST in serum collected from mice induced with APAP. c,d) The levels of TNF‐α and IL‐1β in the hepatic tissues. e,f) The serum levels of CREA and UREA. At 6 h after i.p. stimulation with APAP at 200 mg kg^−1^, mice were treated with different formulations. The PCD NP and TCD groups were treated at the same dose of the PBAP or Tpl unit as that of 1.0 mg kg^−1^ TPCD NP, respectively. In the 10Tpl group, the Tpl dose was tenfold of that contained in TPCD NP. For the Tpl/HMP group, the Tpl dose was the same as that in TPCD NP, while the HMP dose equaled to that generated after complete hydrolysis of TPCD NP. At 12 h after different treatments, animals were euthanized and serum was collected for quantification of biochemical markers. The liver tissues were isolated for quantification of inflammatory mediators. g) H&E‐stained histopathological sections of liver tissues. The lower panel indicates high‐resolution images. Data are mean ± SE (*n* = 6). Statistical significance was assessed by one‐way ANOVA with post hoc LSD tests for data in (a–c, e, f). *P < 0.05, **P < 0.01, ***P < 0.001; ns, no significance. Due to heterogeneity of variance, the Kruskal–Wallis test was used for statistical analysis of data in (d). *P < 0.01, ***P < 0.0001.

Based on the above promising results, we additionally compared therapeutic effects of TPCD NP with PCD NP, TCD, tenfold Tpl, and Tpl/HMP in separate experiments. Similar to the findings in peritonitis and ALI models (Figures 4f–m and 6), treatment of APAP‐challenged mice with TPCD NP achieved desirable protective effects, as implicated by the significantly decreased levels of ALT, AST, TNF‐α, IL‐1β, and H_2_O_2_ as well as CREA and UREA (**Figure** **9**a–f and Figure S13, Supporting Information). For other therapy groups, only some of the examined parameters were significantly reduced. Examination on H&E sections of different groups also demonstrated the more efficaciously protective effect by intervention with TPCD NP (Figure 9g). Collectively, these results demonstrated that, by effectively eliminating ROS, TPCD NP can be used as a nano‐antidote for detoxification of drug‐induced liver and/or kidney injury.

### 2.8 Safety Evaluations of TPCD NP

In view of the above desirable therapeutic results, both in vitro and in vivo experiments were performed to interrogate the safety profile of TPCD NP. Cytotoxicity evaluation was initially examined in RAW264.7 mouse macrophages. After incubation for 6 h, high cell viability was detected regardless of various doses of TPCD NP (Figure S14a, Supporting Information). Even at the dose as high as 1000 µg mL^−1^, the cytotoxicity of TPCD NP in RAW264.7 cells was negligible. Similarly, we found relatively high cell viability after RAW264.7 cells were incubated with various doses of TPCD NP for 12 h (Figure S14b, Supporting Information). In this case, the percentage of viable cells remained above 80% at 1000 µg mL^−1^. Likewise, when a HepG2 human liver cancer cell line was employed, almost no cytotoxicity was found after 6 h of incubation with TPCD NP at different doses (Figure S14c, Supporting Information). Subsequently, the potential risk of hemolysis was examined using fresh erythrocytes isolated from whole blood of Sprague Dawley rats. After incubation with various concentrations of TPCD NP for 2 h, direct observation showed negligible hemolysis (Figure S15a, Supporting Information), although severe hemolysis was observed in the positive control group treated with deionized water. Further quantification revealed that the degree of hemolysis was less than 1% even at a relative high concentration of 2 mg mL^−1^ TPCD NP (Figure S15b, Supporting Information).

Since TPCD NP is expected to be delivered via injection, we performed safety studies after administration via i.p. and i.v. routes. After either i.p. or i.v. injection of TPCD NP at 1000 mg kg^−1^ in Kunming mice, all animals remained healthy and survived, without any behavioral abnormalities such as convulsions, vomiting, and diarrhea. In addition, TPCD NP‐treated mice displayed gradually gained body weight (Figure S16a, Supporting Information), which was similar to the control healthy mice. At day 30 after administration, mice were euthanized. All the treated mice showed the comparable organ index for major organs, including heart, liver, spleen, lung, and kidney (Figure S16b, Supporting Information). Also, complete blood count revealed no distinct variations in typical hematological parameters, such as red blood cell (RBC), hemoglobin (HGB), white blood cell (WBC), and platelet (PLT) (Figure S16c–f, Supporting Information). This in vivo result is in line with the finding on in vitro hemolysis tests (Figure S15, Supporting Information). Furthermore, with the exception of ALT level for the i.v. injection group, we did not find abnormally increased serum levels of typical biomarkers associated with hepatic and renal functions including AST, CREA, and UREA in mice treated with TPCD NP (Figure S16g–j, Supporting Information). Likewise, examination on H&E‐stained sections of major organs from TPCD NP‐administered mice indicated that there were no distinguishable injuries and pathological patterns such as cellular edema, infiltration of inflammatory cells, necrosis, hyperemia, or changes in the morphology of vessels (Figure S16k, Supporting Information). Consequently, these preliminary data indicated that TPCD NP did not cause obvious in vitro cytotoxicity, in vivo systemic toxicity, and inflammatory reactions, and therefore it can serve as a safe nanotherapy.

## 3 Conclusion

In summary, we developed a SOD/catalase‐mimetic material capable of eliminating a broad spectrum of ROS, which can be facilely synthesized by conjugating two functional moieties onto a cyclodextrin scaffold. This pharmacologically active material TPCD can be easily processed into effective antioxidant and anti‐inflammatory nanotherapies. Cellularly, a TPCD‐derived nanotherapy protected macrophages from oxidative stress‐induced apoptosis, after internalization into cells and efficiently scavenging ROS. In several murine models of inflammatory diseases including paw edema, peritonitis, and acute lung injury, the nanotherapy TPCD NP effectively attenuated oxidative stress and inhibited inflammatory responses in diseased tissues or organs. In addition, TPCD NP was able to serve as a nano‐antidote to significantly inhibit drug‐induced liver and/or kidney injury, by eliminating overproduced ROS. In all these cases, treatment with TPCD NP afforded more beneficial outcomes than the corresponding control small‐molecule drug, mainly resulting from its passive targeting capability due to impaired biological barriers at diseased sites of inflammation or injury. Importantly, the broad‐spectrum ROS‐scavenging capacity contributed to the superior efficacy of TPCD NP. In addition, preliminary in vivo studies demonstrated that TPCD NP was safe after delivery via i.p. and i.v. routes at a dose dramatically higher than that used for in vivo therapy. Consequently, TPCD NP deserves further development as an efficacious and safe nanotherapy for treatment of numerous diseases associated with inflammation and oxidative stress. The pharmacological activities of TPCD NP may be further enhanced by surface engineering with functional moieties targeting diseased tissues or mitochondrial oxidative stress, which can also be achieved by packaging with other anti‐inflammatory therapeutics.

## Conflict of Interest

The authors declare no conflict of interest.

## Supporting information

Supplementary
