# Supplementary material for: A Broad‐Spectrum ROS‐Eliminating Material for Prevention of Inflammation and Drug‐Induced Organ Toxicity
Source: Adv Sci (Weinh). 2018 Aug 16;5(10):1800781. doi: 10.1002/advs.201800781 (PMC6193162; doi:10.1002/advs.201800781)
Supplement: Supplementary file 1 — Supplementary [file ADVS-5-1800781-s001.pdf]

Copyright WILEY-VCH Verlag GmbH & Co. KGaA, 69469 Weinheim, Germany, 2018.

Supporting Information

for *Adv. Sci.,* DOI: 10.1002/advs.201800781

A Broad-Spectrum ROS-Eliminating Material for Prevention of Inflammation and Drug-Induced Organ Toxicity

*Lanlan Li, Jiawei Guo, Yuquan Wang, Xiaoxing Xiong, Hui Tao, Jin Li, Yi Jia, Houyuan Hu, and Jianxiang Zhang**

Copyright WILEY-VCH Verlag GmbH & Co. KGaA, 69469 Weinheim, Germany, 2016.

Supporting Information

A Broad-Spectrum ROS-Eliminating Material for Prevention of Inflammation and Drug-Induced Organ Toxicity

*Lanlan Li, Jiawei Guo, Yuquan Wang, Xiaoxing Xiong, Hui Tao, Jin Li, Yi Jia, Houyuan Hu, and Jianxiang Zhang**

Experimental Section

Materials

4-Hydroxy-2,2,6,6-tetramethylpiperidin-1-oxyl (Tempol, defined as Tpl), 1,1-carbonyldiimidazole (CDI), 4-(hydroxymethyl) phenylboronic acid pinacol ester (PBAP), *p*-(hydroxymethyl)phenol (HMP), 4-dimethylaminopyridine (DMAP), anhydrous dimethylformamide (DMF), anhydrous dichloromethane (DCM), anhydrous dimethylsulfoxide (DMSO), acetonitrile (ACN), tetrahydrofuran (THF), carrageen, lipopolysaccharide (LPS, O26:B6), zymosan, 2,2-diphenyl-2-picrylhydrazyl (DPPH), acetaminophen (APAP), and 4',6-diamidino-2-phenylindole (DAPI) were purchased from Sigma-Aldrich (St. Louis, U.S.A.). β-Cyclodextrin (β-CD) and lecithin (from soybean) were obtained from Tokyo Chemical Industry Co., Ltd. (Tokyo, Japan). 1,2-Distearoyl-sn-glycero-3-phosphoethanolamine-N-[methoxy(polyethyleneglycol)-2000 (DSPE-PEG) was provided by CordenPharma (Switzerland). Penicillin, streptomycin, and fetal bovine serum (FBS) were purchased from HyClone (Waltham, U.S.A.). Dulbecco's Modified Eagle Medium (DMEM) was obtained from Gibco (U.S.A.). Cy5 NHS ester (Cy5) and Cy7.5 NHS ester (Cy7.5) were obtained from Lumiprobe, LLC. (U.S.A.). LysoTracker Green (Lyso) was purchased from Invitrogen (U.S.A.). Anti-mouse Ly-6G antibody and anti-mouse CD11b antibody were purchased from BD Biosciences (U.S.A.). Mouse myeloperoxidase (MPO) antibody was purchased from R&D Systems Inc. (Minneapolis, MN). The secondary antibodies Alexa Fluor 568-labeled goat anti-rat IgG and Alexa Fluor 488-labeled donkey anti-goat IgG were obtained from Thermo Fisher Scientific Inc. (Waltham, MA).

Synthesis of a ROS-scavenging material based on β-CD

A ROS-scavenging material (TPCD) was synthesized by sequentially conjugating Tpl and PBAP onto β-CD. First a Tpl-conjugated β-CD (TCD) was synthesized. Specifically, Tpl (1.02 g, 5.9 mmol) was dissolved in anhydrous DCM (10 mL). Then CDI (1.91 g, 11.8 mmol) was added. The reaction was monitored by thin-layer chromatography. After 45 min of reaction, 10 mL of DCM was added into the mixture, followed by washing with 10 mL of deionized water. The organic phase was further rinsed with saturated NaCl solution three times, dried over Na_2SO_4, and concentrated in vacuum. The obtained CDI-activated Tpl (1.65

g) was dissolved in 20 mL anhydrous DMSO, into which β-CD (2.35 g, 2.07 mmol) and DMAP (1.13 g, 9.30 mmol) were added under nitrogen. The obtained mixture was magnetically stirred at room temperature for 24 h. The final product was precipitated from a mixture solution of methanol and diethyl ether, collected by centrifugation, and finally freeze-dried to give a pink powder of TCD.

According to the above mentioned methods, CDI-activated PBAP was synthesized. Briefly, PBAP (1.11 g) and CDI (1.53 g) were dissolved in 15 mL of anhydrous DCM. The mixture was stirred at room temperature for 30 min under nitrogen. The organic phase was washed with deionized water and saturated NaCl solution, dried over Na_2SO_4, and concentrated to obtain CDI-activated PBAP (1.66 g).

Subsequently, 1.66 g CDI-activated PBAP, 1.06 g DMAP, and 0.42 g TCD were co-dissolved in 20 mL of anhydrous DMSO under nitrogen. The reaction was carried out for 48 h at room temperature. The product was obtained by precipitation from 80 mL of deionized water, and collected by centrifugation. After thorough rinsing with water, the sample was collected by lyophilization to give a light pink powder of TPCD.

Though the similar procedures, PBAP-conjugated β-CD (PCD) was synthesized.[1]

Characterization of materials

^{1}H NMR spectra were recorded on a spectrometer operating at 600 MHz (DD2, Agilent). Matrix-assisted laser desorption/ionization time-of-flight (MALDI-TOF) mass spectrometry measurement was performed with a Waters Micromass TofSpec-2E operated in a linear mode. Fourier transform infrared (FT-IR) spectra were recorded on a PerkineElmer FT-IR spectrometer (100S). UV-visible spectroscopy was conducted on an ultraviolet spectrophotometer (TU-1901, Beijing Purkinje General instrument, China). Electron paramagnetic resonance (EPR) spectroscopy was carried out at room temperature on a JES-FA200 EPR spectrometer (Japanese Electronics Co., LTD) operating at 9.4 GHz with a 100.00 kHz magnetic field modulation.

Fabrication of nanoparticles based on TPCD

TPCD nanoparticles (TPCD NPs) were prepared by a nanoprecipitation/self-assembly method.[2] In brief, lecithin (6 mg) and DSPE-PEG (9 mg) were dissolved in 0.6 mL of ethanol, into which 15 mL of deionized water was added. The obtained mixture was heated to 65°C for 1 h. Then, the methanol solution (5 mL) containing 50 mg TPCD was added dropwise into the preheated aqueous solution of lipid, followed by incubation for 2 h at room temperature. The remaining organic solvent was removed by vacuum evaporation. Finally, TPCD NP was obtained by freeze-drying. In addition, different solvent combinations were used to dissolve TPCD in order to prepare TPCD NPs with different sizes. Through similar procedures, TPCD NPs containing Cy5 or Cy7.5 were fabricated.

Characterization of nanoparticles

The size, size distribution profiles, and zeta-potential values of NPs were measured using a Malvern Zetasizer NanoZS instrument at 25°C. The morphology of NPs was observed with transmission electron microscopy (TEM) using a TECNAI-10 microscope (Philips, Netherlands), operating at an acceleration voltage of 80 kV. Samples were prepared by dipping the formvar-coated Cu grid into aqueous solution of NPs. Extra solution was evaporated at room temperature. Scanning electron microscopy (SEM) was also employed to observe the morphology of TPCD NP by a FIB-SEM microscope (Crossbeam 340, Zeiss).

Hydrolysis of TPCD in the presence of hydrogen peroxide
After TPCD was incubated with an aqueous solution containing 1.0 mM H_2O_2, the hydrolyzed products were collected by lyophilization and characterized by ^{1}H NMR spectroscopy and MALDI-TOF mass spectrometry.

The hydrolyzed products were also analyzed by high performance liquid chromatography (HPLC). To this end, a predetermined amount of TPCD was mixed with various concentrations of H_2O_2 at 37°C for 24 h. Then the hydrolyzed samples were analyzed with a Shimadzu high-performance liquid chromatography (HPLC) system (LC-2030C and RF-20A), using a methanol-water eluent (20:80, v:v) at a flow rate of 1.0 mL/min.

Elimination of radical by TPCD
The free radical scavenging capability of TPCD was measured using a previously established protocol with minor modifications. Briefly, 1.5 mL of a fresh solution of DPPH• (100 μg/mL) was incubated in 3 mL of methanol containing different concentrations of TPCD (from 0, 0.063, 0.125, 0.25, 0.5, 1.0, to 2.0 mg/mL) for 30 min in dark. Subsequently, the absorbance at 517 nm was recorded by UV-visible spectroscopy, and the DPPH median elimination concentration (EC_{50}) values were calculated. Based on the similar methods, the radical scavenging capability of different materials (including TPCD, TCD, and PCD) was quantified and compared.

Scavenging of superoxide anion and H_2O_2 by TPCD
To examine the superoxide anion-eliminating capability of TPCD, different concentrations of TPCD (varying from 0.0016, 0.008, 0.04, 0.20, to 1.0 mg/mL) were incubated with an excess amount of superoxide anion. The remaining superoxide anion was measured by the Superoxide Anion Free Radical Detection Kit (Nanjing Jiancheng Bioengineering Institute, China). Of note, the superoxide anion was generated by the reaction of xanthine with xanthine oxidase. Finally, the superoxide anion-eliminating capacity of TPCD was quantified by measuring the absorbance at 550 nm.

In separate experiments, various concentrations of TPCD (from 0.25, 1.0, 2.0, to 4.0 mg/mL) were incubated in 2 mL of PBS containing 50 mM H_2O_2 at 37°C for 24 h. Using the Hydrogen Peroxide Detection Kit (Nanjing Jiancheng Bioengineering Institute, China), the concentration of remaining H_2O_2 was determined by measuring the absorbance at 405 nm, and the H_2O_2-eliminating capacity was calculated.

Through the similar procedures, the superoxide anion or H_2O_2-eliminating capability of TCD and PCD was measured.

Scavenging of hypochlorite by TPCD

The hypochlorite scavenging capability by TPCD was determined by using a luminescent nanoprobe (Lu-bCD NP) developed in our previous study.[3] First, the standard curve was established using Lu-bCD NP as a probe. Specifically, 50 μL of aqueous solution containing Lu-bCD NP (10 mg/mL) was reacted with 50 μL NaClO solution with concentrations varying from 0, 0.625, 1.25, 2.5, 5.0,10, 50, to 100 mM in a black 96-well plate. Immediately after mixing, luminescence imaging was performed by an IVIS Spectrum system. The instrument parameters were set as follows: exposure time = 5 min, f/stop = 1, binning = 8, and no optical filter. The standard curve was established by plotting the chemiluminescence intensity as a function of NaClO concentrations. Subsequently, Lu-bCD NP was employed as the probe for final quantification of the hypochlorite scavenging capability. Briefly, 25 μL of aqueous solution containing different concentrations of TPCD (varying from 0, 0.4, 1.0, 4.0, to 10 mg/mL) was incubated with 475 μL of aqueous solution containing 100 mM NaClO at room temperature. After 15 min, 50 μL of the supernatant was reacted with 50 μL aqueous solution containing 10 mg/mL Lu-bCD NP and immediately imaged by an IVIS Spectrum system. Then the residual ClO^- was calculated according to the standard curve. Through the similar procedures, the hypochlorite scavenging capability of different materials (including TPCD, TCD, and PCD) was determined and compared.

In vitro ROS-responsive hydrolysis of TPCD NPs

For in vitro hydrolysis tests, 5 mg freshly produced NPs were incubated in 5 mL PBS (0.01 M, pH 7.4) containing various concentrations of H_2O_2 (varying from 0.01, 0.05, 0.25, 0.5, to 1.0 mM) at 37°C. At predetermined time points, the transmittance values of NP-containing aqueous solutions (1.0 mg/mL) were measured at 500 nm. The hydrolysis degree was calculated.

To characterize the H_2O_2-concentration dependent change in size and morphology of TPCD NP, 5 mg TPCD NP was separately incubated in PBS conainting 0.1, 0.25, or 1.0 mM H_2O_2 at 37°C for 3 h. Then the morphology, particle size distribution profiles, and zeta-potenial values of the corresponding samples were characterized by TEM and DLS.

Cytotoxicity evaluation by MTT assay

A human hepatocellular liver carcinoma HepG2 cell and a RAW264.7 mouse macrophage cell were cultured in 96-well plates at a density of 1.0×10^4 cells per well in 100 μL DMEM containing 10% (v/v) FBS, 100 U/mL of penicillin, and 100 mg/mL of streptomycin. Cells were incubated at 37°C in a humidified atmosphere containing 5% CO_2 before additional experiments. After 24 h, cells were treated with TPCD NP at different doses for various time periods. The cell viability was quantified by MTT assay.

In vitro cellular uptake

RAW264.7 macrophage cells were seeded onto sterilized glass coverslips in 12-well plates at a density of 1.0 × 10^5 cells per well. After incubation in 1 mL of growth medium for 12 h, the culture medium was replaced with 1 mL of fresh medium containing Cy5-labeled TPCD NP (Cy5/TPCD NP) at 2 µg/mL of Cy5 and incubated for different periods of time. Before observation, LysoTracker Green (75 nM) was added and cells were incubated for additional 2 h. Cells were washed with PBS, fixed with 4% paraformaldehyde, and stained with DAPI. Confocal laser scanning microscopy (CLSM) observation was conducted by a confocal microscope (Leica, Heidelberg, Germany).

To quantify internalized TPCD NP, RAW264.7 cells were seeded in a 12-well plate at a density of 3.0 × 10^5 cells per well in 1 mL of growth medium for 24 h. Then the culture medium was replaced with 1 mL of fresh medium containing Cy5/TPCD NP at 2 µg/mL of Cy5. After incubation for various periods of time, cells were digested and fluorescence intensity was determined via fluorescence activated cell sorting (FACS) by flow cytometry (FACSCalibur, Becton Dickinson, U.S.A.). Through similar procedures, the dose-dependent internalization profile was examined after cells were incubated with varied doses of Cy5/TPCD NP for 2 h.

In vitro anti-apoptosis activity of TPCD NP in macrophages

RAW264.7 cells were seeded in a 12-well plate at a density of 3 × 10^5 cells/well and incubated overnight. Cells were incubated with TPCD NP at 100 µg/mL for 2 h, and then the culture medium was replaced with 1 mL of fresh medium containing 200 µM H_2O_2. After 24 h of incubation, cells were washed with a cold BioLegend's cell staining buffer, collected by centrifugation, and resuspended in an Annexin V binding buffer containing Annexin V-APC (Annexin V) and propidium iodide (PI) solution. After cells were vortexed gently and incubated in a dark room for 15 min, flow cytometry analysis was performed immediately.

According to the similar procedures, PCD NP (with the same dose of the PBAP unit as that of 100 µg/mLTPCD NP), TCD (with the same dose of theTpl unit as that of 100 µg/mL TPCD NP), high and normal dose of Tpl, and combined use of Tpl/HMP were served as different therapy controls. For the 10Tpl group, the Tpl dose was 10-fold of that contained in TPCD NP. In the Tpl/HMP group, the Tpl dose was the same as that in TPCD NP, while the dose of HMP equals to that produced after complete hydrolysis of TPCD NP.

Animals

All the animal care and experimental protocols were performed in compliance with the Animal Management Rules of the Ministry of Health of the People's Republic of China (No. 55, 2001) and the guidelines for the Care and Use of Laboratory Animals of Army Medical University (Chongqing, China). BALB/c mice (18-22 g), C57BL/6 mice (18-22 g), Kunming mice (18-22 g), and Sprague Dawley rats (180-200 g) were obtained from the Animal Center of the Army Medical University. Animals were housed in standard cages under optical light,

temperature, and humidity conditions, with *ad libitum* access to water and food. All the animals were acclimatized for one week before further experiments.

Hemolysis testing

Blood samples were collected from male Sprague Dawley rats, from which 2% erythrocytes suspension in saline was prepared. The erythrocyte suspension was then mixed with TPCD NP at different final concentrations (varied from 0.0032, 0.016, 0.08, 0.4, to 2.0 mg/mL), with the total volume of 1 mL. In the positive control group, deionized water was added, while saline solution was added in the negative group. After the different suspensions were incubated at room temperature for 2 h, all samples were centrifuged at 500*g* for 5 min. The optical density (OD) of supernatant was measured at 570 nm, with OD at 620 nm serves as a reference. The hemolytic degree was calculated according to the follow equation.

$$\text{Hemolysis percentage (\%)} = \frac{\text{Sample OD}_{570-620} - \text{Negative control OD}_{570-620}}{\text{Positive control OD}_{570-620} - \text{Negative control OD}_{570-620}} \times 100$$

Acute toxicity evaluation of TPCD NP in mice

Male Kunming mice (18-23 g) were randomly assigned into three groups (n = 6). Mice in the TPCD NP group were intravenously (i.v.) or intraperitoneally (i.p.) administered with TPCD NP at 1000 mg/kg, while healthy mice without any treatments served as the control group. After different treatments, mice were weighed at defined time points. Their behaviors were monitored for any signs of illness each day. At day 30, animals were euthanized, and blood samples were collected for hematological analysis (Sysmex KX-21, Japan). Major organs including heart, liver, spleen, lung, and kidney were isolated, weighed, and histological sections were prepared and stained with hematoxylin and eosin (H&E).

Treatment of carrageen-induced paw edema in mice

Acute inflammation in the paw of Sprague Dawley rats was induced by intradermal (i.d.) injection of 100 μL of saline containing 2 wt% carrageen in the left hind paw.[4] Then mice were randomly assigned into four groups (n = 5). TPCD NP at 1.0 mg/kg, TPCD NP at 5.0 mg/kg, Tpl, or saline was separately injected in the left hind foot pad at 30 min after carrageen stimulation. TPCD NP at 5.0 mg/kg contains the same dose of the Tpl unit as that of the Tpl group. The paw volume was measured before and after carrageen administration using a transducer-linked plethysmometer (YLS-7B, Yiyan technology development Co., Ltd., Jinan, China). The edema degree was calculated according to the follow equation.

$$\text{Edema degree (ED) \%} = \frac{V_t - V_0}{V_0} \times 100\%$$

Where V_0 and V_t represent the paw volume before and after inflammation, respectively.

Treatment of peritonitis in mice

Peritonitis in male BALB/c mice was induced by i.p. injection of 1 mL zymosan (1 mg/mL),[3] while the control mice were treated with saline. In each group (n = 6), mice were i.p. administered with various doses of TPCD NP (0.1 or 1.0 mg/kg) or free Tpl in each mouse at 1 h after induction of peritonitis. Of note, TPCD NP at 1.0 mg/kg had the same dose of the Tpl unit as that of the Tpl group. At 6 h after different treatments, mice were euthanized. The peritoneal lavage was collected, into which 500 μL of Triton X-100 (0.3 wt.%) was added. The mixture was homogenized with a tissue-tearor homogenizer (BioSpec Products Inc., U.S.A.) in an ice bath for 5 s three times, with an interval 30 s. Subsequently, the samples were centrifuged at 16000*g* for 10 min at 4°C. The levels of myeloperoxidase (MPO), H_2O_2, tumor necrosis factor-α (TNF-α), and interleukin-1β (IL-1β) in the supernatant were quantified by ELISA assay.

Following exactly the similar protocols, therapeutic effects of PCD NP, TCD, 10-fold Tpl, and Tpl/HMP were also investigated in a seperated study. For all the control groups, the dosing regimens were the same as those employed in the in vitro anti-apoptotic experiment as aforementioned.

Therapeutic effects of TPCD NP in mice with LPS-induced acute lung injury

To induce acute lung injury (ALI) in BALB/c mice, 50 μL of PBS containing LPS at 1 mg/mL was administered by intratracheal (i.t.) inoculation.[3] Then mice were randomly assigned into four groups (n = 6). At 1 h after stimulation with LPS, saline was i.v. injected in the model group, while TPCD NP at 0.1 or 1.0 mg/kg and Tpl with the same dose of the Tpl unit as that of 1 mg/kg TPCD NP was separately administered by i.v. injection in other groups. In another normal control group, healthy mice were treated with saline. At 11 h after different treatments, mice were euthanized. The mouse trachea was exposed and a small horizontal incision was made. Then, 1.0 ml PBS was injected into the lungs, and bronchoalveolar lavage fluid was collected immediately. After three times of lavage, 1 mL of lavage fluid was withdrawn and incubated with ACK lysis buffer for 5 min to deplete red blood cells. Subsequently, cells were stained with V450-conjugated rat anti-mouse CD11b and PE-conjugated rat anti-mouse Ly-6G for analysis by flow cytometry. In addition, 500 μL Triton X-100 (0.3 wt%) was added into 1.5 ml of residual bronchoalveolar lavage fluid and homogenized, followed by centrifugation at 16000*g* for 10 min at 4°C. The levels of MPO, H_2O_2, TNF-α, and IL-1β in the supernatant were assessed by ELISA.

In a separate study, the severity of pulmonary edema was evaluated by calculating the wet/dry weight ratio of lung tissues. To this end, the lungs were separated and weighed immediately to obtain the wet weight. Then the tissues were dried at 60°C for 48 h to obtain the data of dry weight. Histological sections were also made and stained with H&E.

To evaluate the efficacies of PCD NP, TCD, 10-fold Tpl, and Tpl/HMP, another cohort of studies were also conducted according to the above mentioned procedures. The dosing regimens were same as those used in in vitro anti-apoptotic experiments.

Blood circulation and in vivo targeting of TPCD NP in ALI mice

The mouse model of ALI was established according to the aforementioned method. After 1 h, Cy7.5-labeled TPCD NP was i.v. administered through the tail vein at 20 µg of Cy7.5 in each mouse. Meanwhile, saline was injected in the control mice. At each predetermined time points, blood and major organs were collected. Ex vivo imaging was carried out with an IVIS Spectrum living imaging system (PerkinElmer, U.S.A.), and the fluorescence intensity was analyzed by the Living Imaging software.

Therapeutic effects of TPCD NP in mice with APAP-induced hepatotoxicity and renal injury

Male C57BL/6 mice were fasted for 15 h before experimentation. The APAP solution was prepared by dissolving APAP in sterilized saline at 20 mg/mL.[5] Mice were i.p. injected with APAP at 200 mg/kg and randomly divided into 4 groups (n = 6). At 6 h after stimulation with APAP, saline was i.v. injected in the model group, while TPCD NP (at 0.1 or 1.0 mg/kg) or free Tpl (with the same dose of the Tpl unit as that of 1.0 mg/kg TPCD NP) was separately administered by i.v. injection in other groups. In another normal control group, healthy mice received saline alone. At 12 h after different treatments, mice were euthanized. Blood samples were collected for hematological analysis. In addition, the livers were isolated and weighed. A part of liver tissues was fixed in paraformaldehyde for histopathological analysis. The remaining livers were homogenized in cold PBS with a tissue-tearor homogenizer and then centrifuged at 16,000*g* for 10 min at 4°C. The resulting supernatants were collected for quantification of the levels of H_2O_2, TNF-α, and IL-1β by ELISA.

In addition, the neutrophil counts and MPO levels in livers were analyzed by immunofluorescence. In brief, replicate sections were deparaffinized and blocked with 1% BSA and 0.3% Triton X-100 for 30 min at room temperature. Then the sections were incubated with antibodies to Ly-6G and MPO at 37°C for 30 min. After 24 h, the slices were incubated with the secondary antibody Alexa Fluor 568-labeled goat anti-rat IgG (for labeling of neutrophils) and Alexa Fluor 488-labeled donkey anti-goat IgG (for labeling of MPO) for 50 min. After the nuclei were stained with DAPI, the sections were imaged by CLSM.

Finally, in vivo therapeutic benefits of TPCD NP were compared with different control therapies, including PCD NP, TCD, 10-fold Tpl, and Tpl/HMP, based on the similar therapeutic procedures as mentioned above. The dosing regimens were exactly the same as those used for in vitro anti-apoptotic experiments.

In vivo liver targeting in mice with APAP-induced organ toxicity by TPCD NP

Drug-induced liver injury in mice was induced by i.p. injection of APAP as mentioned above. After 6 h of stimulation, animals were administered with Cy7.5-labeled TPCD NP at 20 µg of Cy7.5 in each mouse by i.v. injection. In the control group, mice were treated with i.v. injection of saline. At 12 h after different treatments, mice were euthanized. The livers and other major organs were excised for ex vivo imaging with an IVIS Spectrum living imaging system.

Statistical analysis

All data are expressed as mean ± standard error of the mean (SE). Statistical analyses were performed with the software of SPSS12 using one-way ANOVA with post-hoc LSD tests for experiments consisting of more than two groups, and unpaired *t*-test was performed for experiments with two groups. Statistical significance was assessed at $P < 0.05$. For data with heterogeneity of variance, the Kruskale-Wallis test was used for statistical analysis.

References

[1] Q. Zhang, F. Zhang, Y. Chen, Y. Dou, H. Tao, D. Zhang, R. Wang, X. Li, J. Zhang, *Chem. Mater.* **2017,** *29*, 8221.
[2] D. Zhang, Y. Wei, K. Chen, X. Zhang, X. Xu, Q. Shi, S. Han, X. Chen, H. Gong, X. Li, J. Zhang, *Adv. Healthc. Mater.* **2015,** *4*, 69.
[3] J. Guo, H. Tao, Y. Dou, L. Li, X. Xu, Q. Zhang, J. Cheng, S. Han, J. Huang, X. Li, X. Li, J. Zhang, *Mater. Today* **2017,** *20*, 493.
[4] L. Che, J. Zhou, S. Li, H. He, Y. Zhu, X. Zhou, Y. Jia, Y. Liu, J. Zhang, X. Li, *Int. J. Pharm.* **2012,** *439*, 307.
[5] P. K. Barman, R. Mukherjee, B. K. Prusty, S. Suklabaidya, S. Senapati, B. Ravindran, *Cell Death Dis.* **2016,** *7*, e2224.

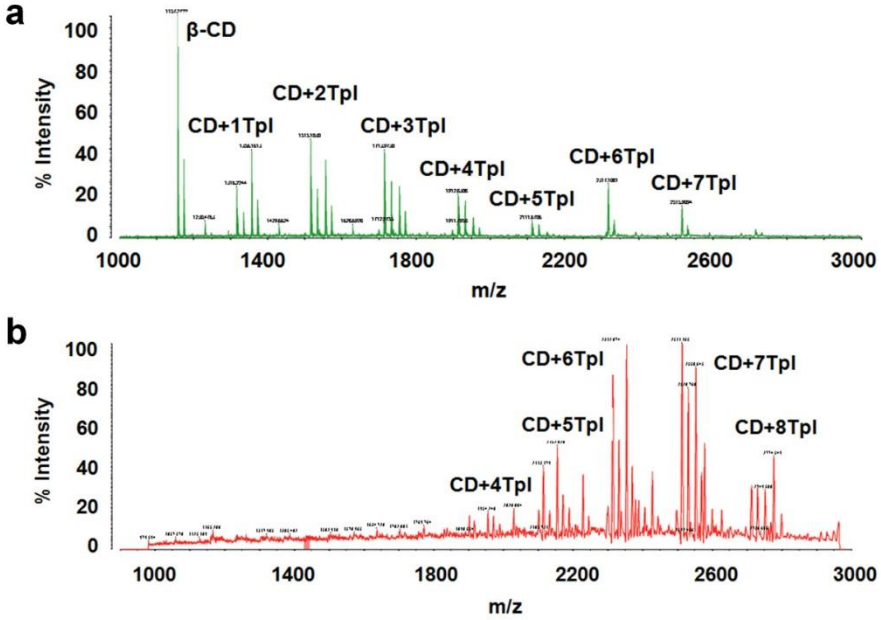

Figure S1. MALDI-TOF mass spectra of TCD before (a) and after (b) thorough washing with deionized water.

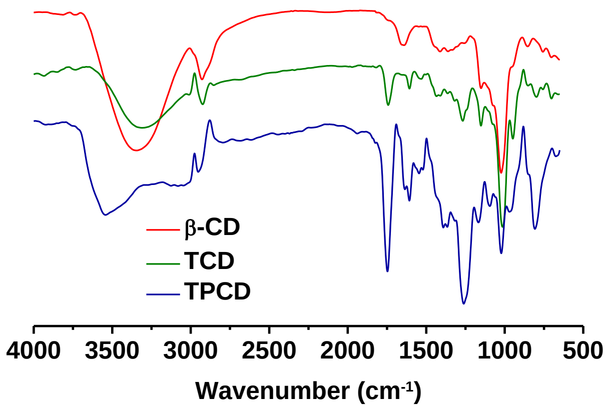

Figure S2. FT-IR spectra of β-CD, TCD, and TPCD.

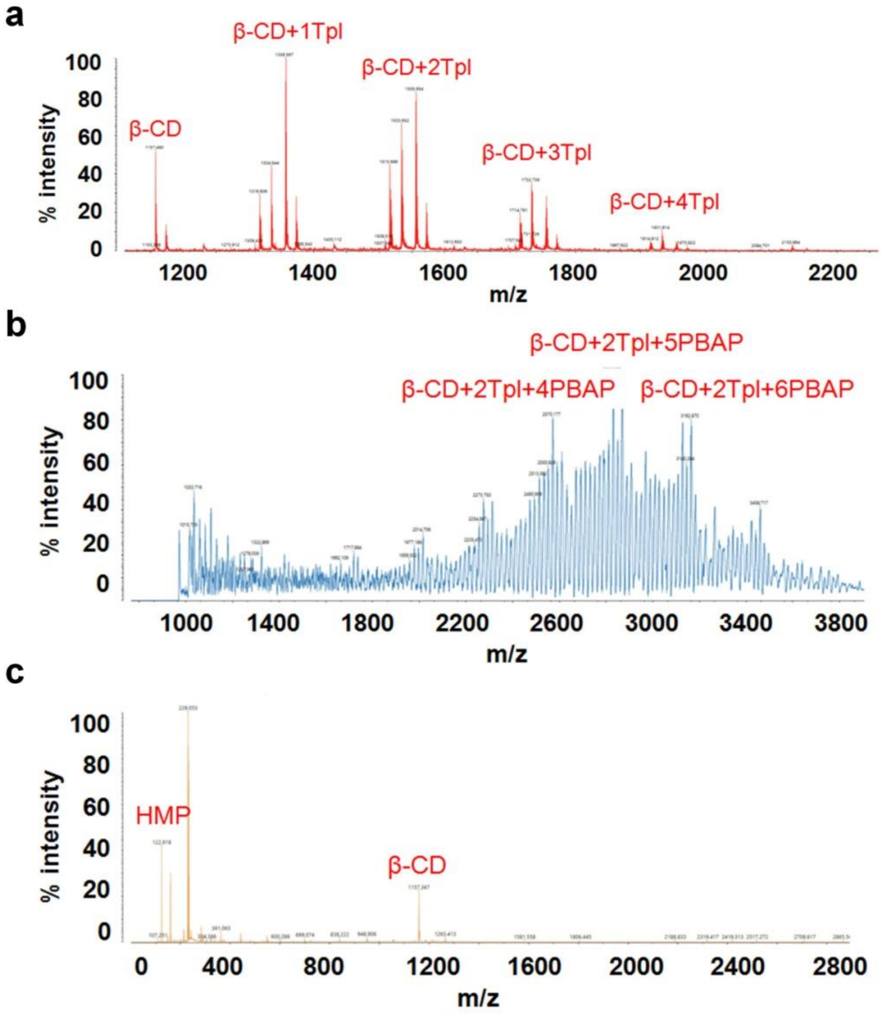

Figure S3. Characterization of different materials by matrix-assisted laser desorption/ionization time-of-flight (MALDI-TOF) mass spectroscopy. (a-b) MALDI-TOF mass spectra of TCD (a) and TPCD (b). (c) The MALDI-TOF mass spectrum of the hydrolyzed products of TPCD in 1 mM of H_2O_2.

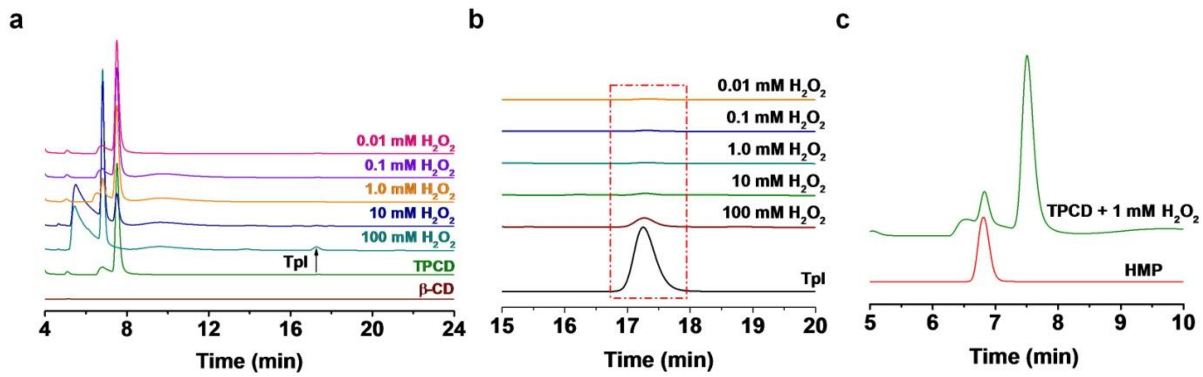

Figure S4. HPLC analysis of TPCD hydrolysis in the presence of H_2O_2. (a) The HPLC traces of hydrolyzed products after 4 mg/mL TPCD was incubated with various concentrations of H_2O_2 at 37°C for 24 h. The samples were analyzed by a methanol-water eluent (20:80, v:v) at a flow rate of 1.0 mL/min. TPCD alone and β-CD were used as controls. (b) The HPLC curves of Tpl and the hydrolyzed samples after 4 mg/mL of TPCD was incubated with various concentrations of H_2O_2 at 37°C for 24 h. (c) The HPLC chromatograms of HMP and the hydrolyzed products after 4 mg/mL TPCD was incubated with 1 mM H_2O_2 at 37°C for 24 h.

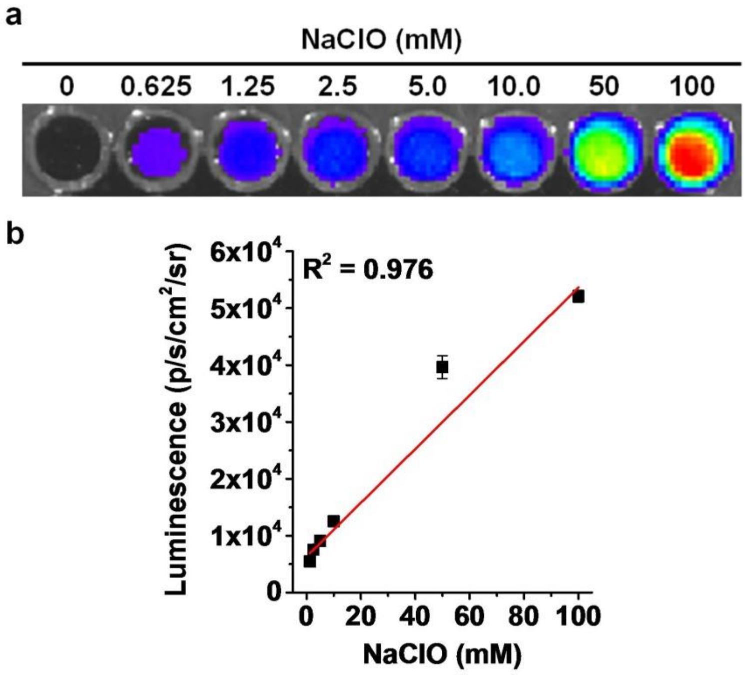

Figure S5. The standard curve for quantification of eliminated ClO^- using a luminescent nanoprobe Lu-bCD NP. (a) Ex vivo luminescence images of 10 mg/mL Lu-bCD NP after incubation with various concentrations of NaClO. (b) A standard curve indicating the luminescent intensities of Lu-bCD NP as a function of NaClO concentrations, which was employed for quantification of ClO^--scavenging capability by different materials. Data are mean ± SE (n = 3).

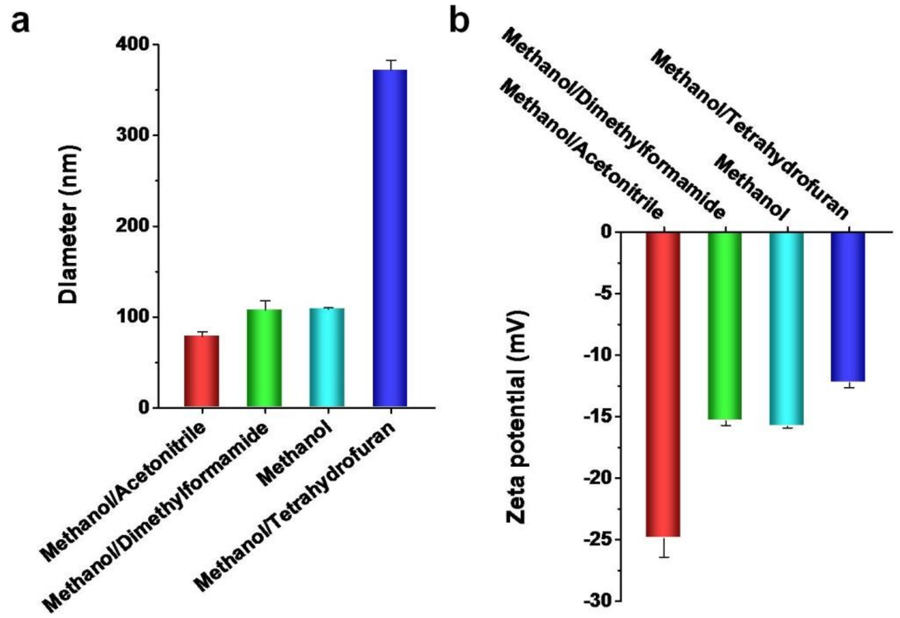

Figure S6. Characterization of TPCD NPs prepared using different solvent mixtures. (a-b) The mean diameter (a) and zeta-potential values (b) of TPCD NPs prepared using different solvents. Data are mean ± SE (n = 3).

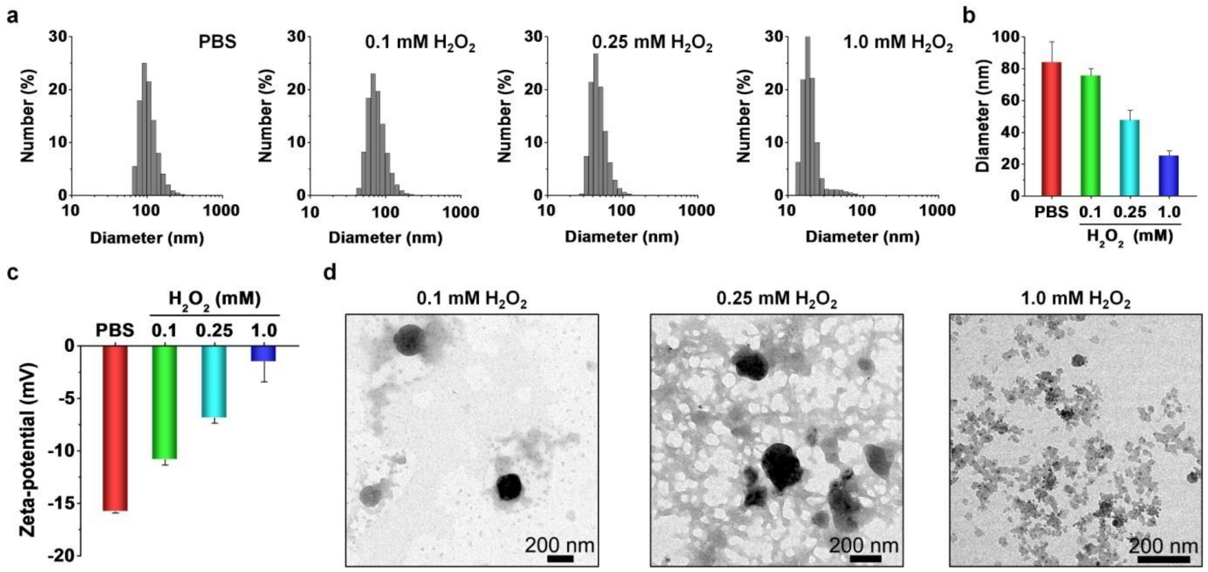

Figure S7. The H_2O_2 concentration-dependent hydrolysis of TPCD NP. (a-c) Changes in size distribuiton profiles (a), mean diameter (b), and zeta-potential values (c) of TPCD NP after incubation in PBS or PBS containing different concentrations of H_2O_2 for 3 h. (d) TEM images of TPCD NP samples after hydrolysis in various concentrations of H_2O_2. Data in (b-c) are mean ± SE (n = 3).

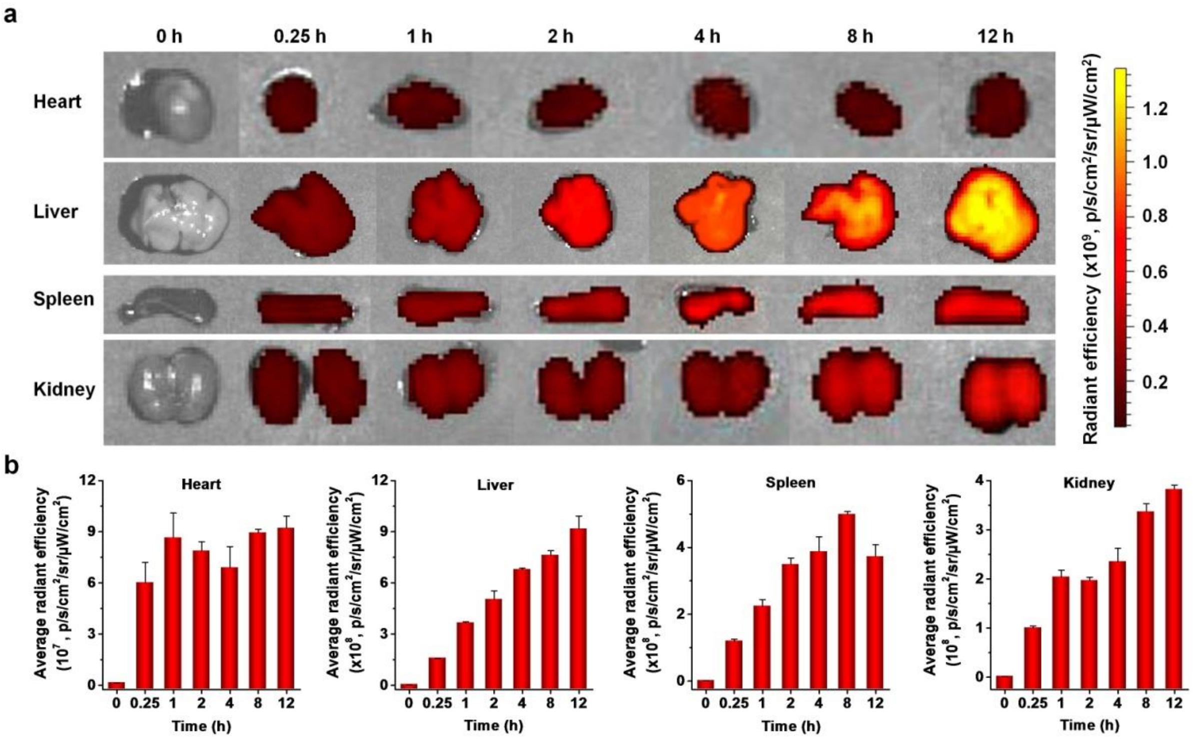

Figure S8. Accumulation of Cy7.5/TPCD NP in typical major organs. (a-b) Ex vivo images (a) and quantitative analysis data (b) show the distribution of Cy7.5/TPCD NP in heart, liver, spleen, and kidney of ALI mice after i.v. administration for different periods of time. Data are mean ± SE (n = 3).

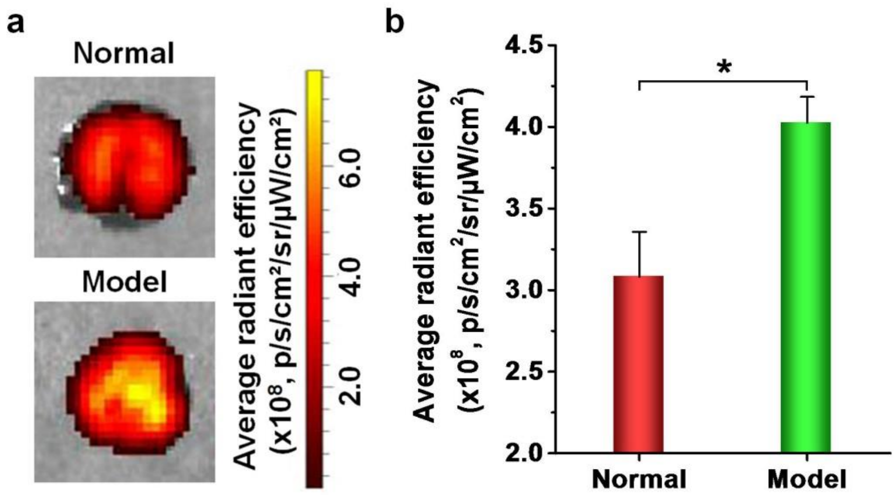

Figure S9. Pulmonary targeting of i.v. administered Cy7.5/TPCD NP in ALI mice. (a-b) Representative ex vivo images (a) and quantitative analysis (b) of the accumulation of Cy7.5/TPCD NP in the lungs of normal or ALI mice at 12 h after i.v. administration. Data are mean ± SE (n = 3). *P < 0.05.

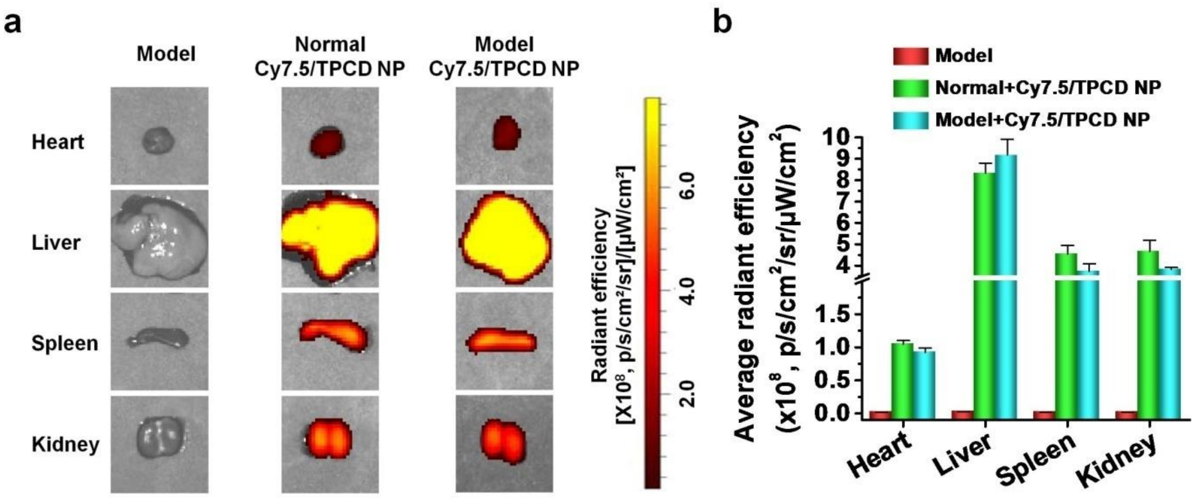

Figure S10. Distribution of i.v. administered Cy7.5/TPCD NP in major organs from ALI mice. (a-b) Representative ex vivo images (a) and quantitative data (b) shwoing the accumulation of Cy7.5/TPCD NP in the heart, liver, spleen, and kidney of normal or ALI mice at 12 h after i.v. administration. Data are mean ± SE (n = 3).

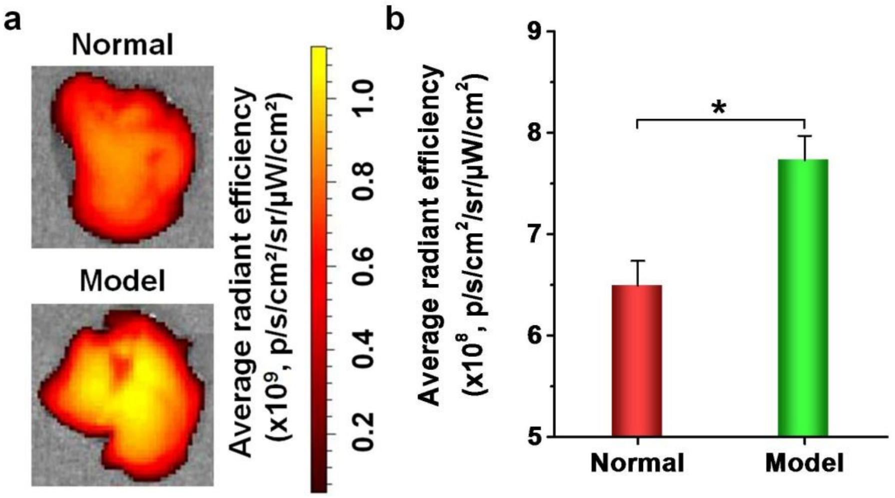

Figure S11. Accumulation of i.v. administered Cy7.5/TPCD NP in the liver of normal mice or mice with APAP-induced liver injury. (a-b) Ex vivo images (a) and quantitative analysis (b) of the accumulation of Cy7.5/TPCD NP in the liver of normal mice or APAP-stimulated mice at 12 h after i.v. administration. Data are mean ± SE (n = 3). *$P < 0.05$.

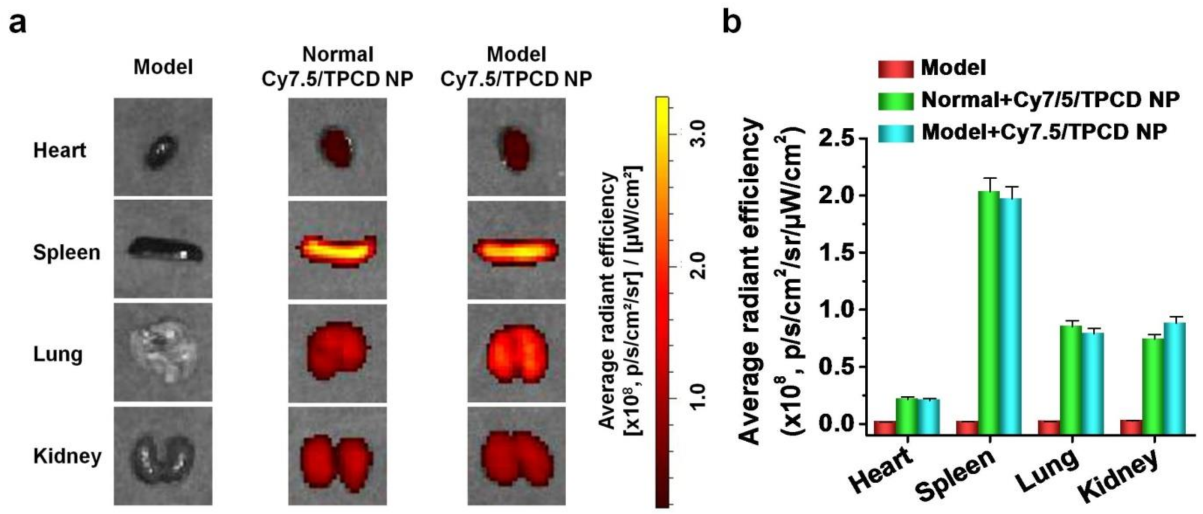

Figure S12. Distribution of i.v. administered Cy7.5/TPCD NP in major organs of normal mice or mice with APAP-induced liver injury. (a-b) Ex vivo images (a) and quantitative data (b) shwoing the accumulation of Cy7.5/TPCD NP in the heart, spleen, lung, and kidney of normal mice or APAP-stimulated mice at 12 h after i.v. administration. Data are mean ± SE (n = 3).

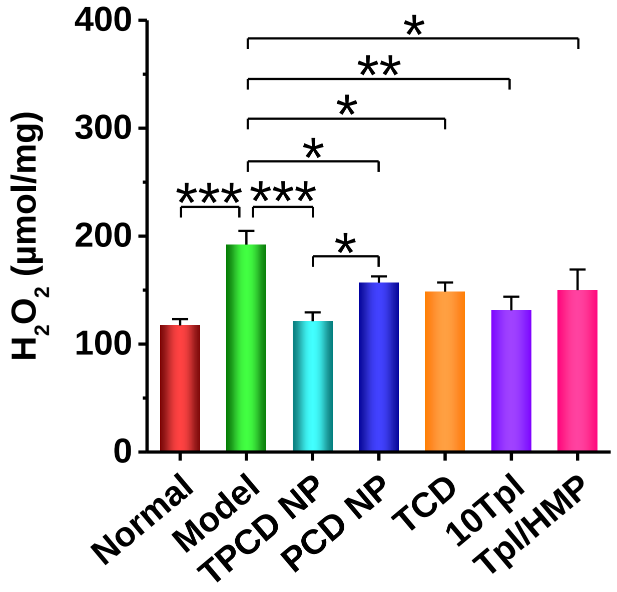

Figure S13. The expression levels of H_2O_2 in the hepatic tissues of mice with or without APAP-induced injury and after treatment with different therapies. At 6 h after i.p. stimulation with APAP at 200 mg/kg, mice were treated with different formulations. The PCD NP and TCD groups were treated with the same dose of the PBAP or Tpl unit as that of 1.0 mg/kg TPCD NP, respectively. In the 10Tpl group, the Tpl dose was 10-fold of that contained in TPCD NP. For the Tpl/HMP group, the Tpl dose was the same as that in TPCD NP, while the HMP dose equaled to that generated after complete hydrolysis of TPCD NP. At 12 h after different treatments, animals were euthanized and the liver tissues were isolated for quantification of H_2O_2. Data are mean ± SE (n = 6). Statistical significance was assessed by one-way ANOVA with post-hoc LSD tests. $*P < 0.05$, $**P < 0.01$, $***P < 0.001$.

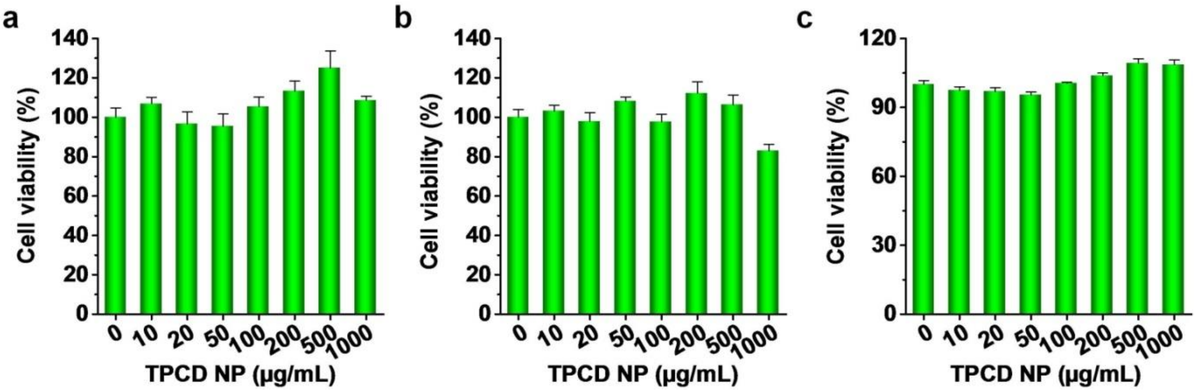

Figure S14. In vitro cytotoxicity evaluations on TPCD NP. (a-b) Cell viability of RAW264.7 mouse macrophages after incubation with various concentrations of TPCD NP for 6 h (a) and 12 h (b). (c) Viability of HepG2 human liver cancer cells after incubation with various doses of TPCD NP for 6 h. Data are mean ± SE (n = 6).

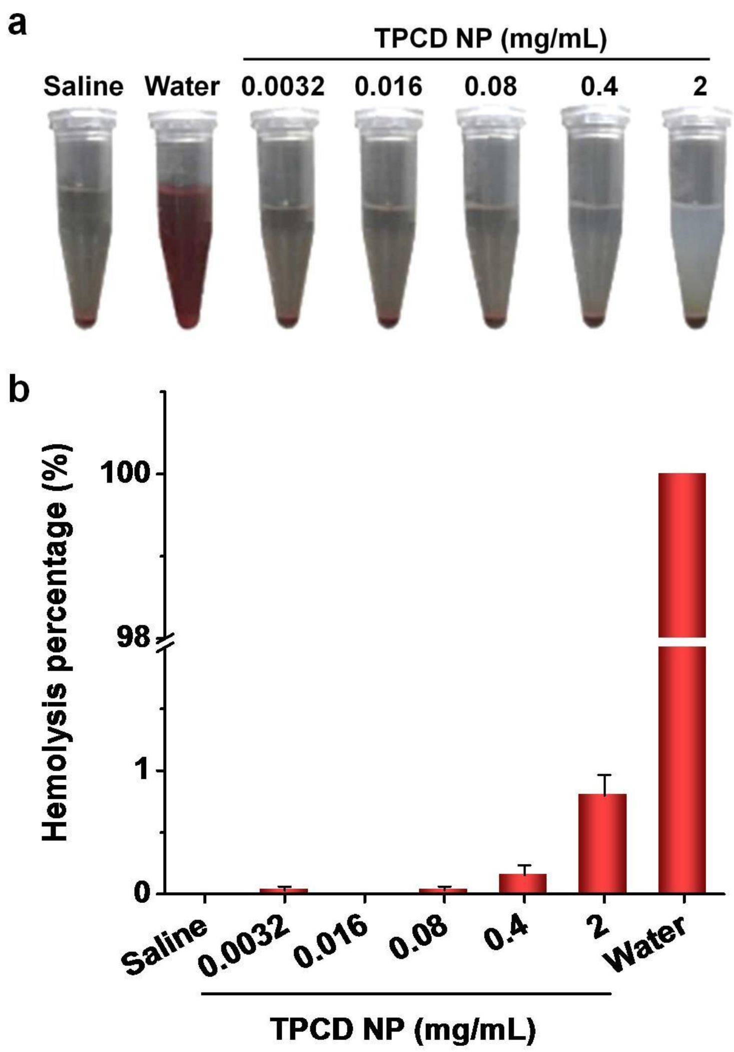

Figure S15. Hemolysis evaluation on TPCD NP using fresh erythrocytes collected from Sprague Dawley rats. (a) Representative digital photos showing erythrocytes that were mixed with saline (negative control), TPCD NP at various concentrations (ranging from 0.0032, 0.016, 0.08, 0.4, to 2.0 mg/mL), or pure water (positive control) and incubated at room temperature for 2 h. (b) The hemolysis degrees of various groups calculated by quantification of absorbance at 500 nm due to hemoglobin. Data are mean ± SE (n = 3).

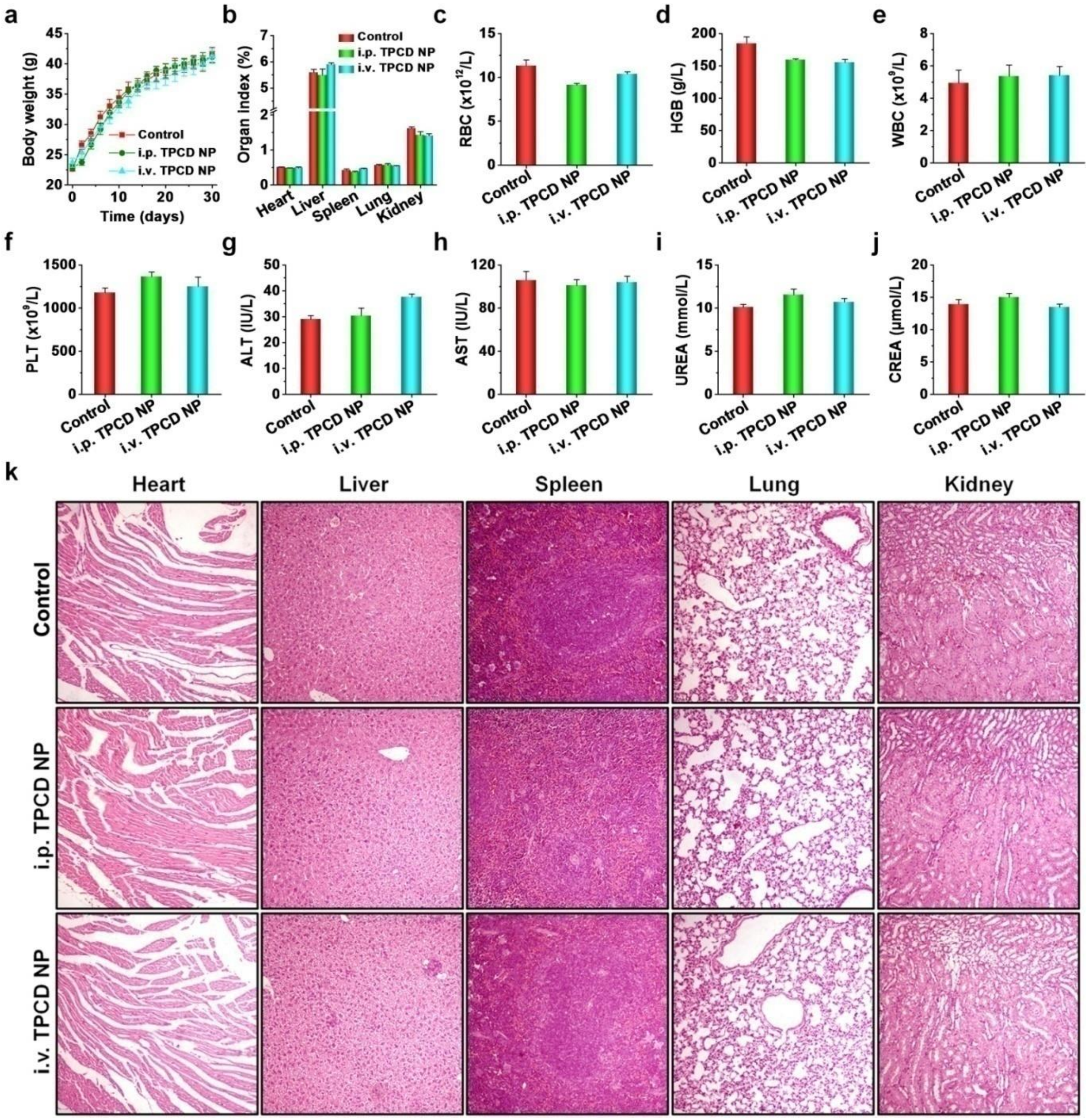

Figure S16. Acute toxicity evaluation of TPCD NP after i.p. or i.v. administration at 1000 mg/kg. (a) Changes in the body weight of normal mice (the control group) or mice treated with TPCD NP administered via i.p. or i.v. injection. (b) The organ index of mice at day 30 after different treatments. (c-f) The levels of representative hematological parameters including red blood cell (RBC), hemoglobin (HGB), white blood cell (WBC), and platelet (PLT). (g-j) Typical biochemical markers related to hepatic and renal functions, including ALT, AST, UREA, and CREA. (k) H&E-stained histopathological sections of various organs resected from mice subjected to different treatments. Data are mean ± SE (n = 6).
